# The neonatal Fc receptor (FcRn) is required for porcine reproductive and respiratory syndrome virus uncoating

**DOI:** 10.1128/jvi.01218-24

**Published:** 2024-12-09

**Authors:** Kang Yang, Jiarui Dong, Jian Li, Rui Zhou, Xiangchao Jia, Zhijian Sun, Weida Zhang, Zili Li

**Affiliations:** 1 State Key Laboratory of Agricultural Microbiology, Key Laboratory of Preventive Veterinary Medicine in Hubei Province, College of Veterinary Medicine, Huazhong Agricultural University 47895 , Wuhan, Hubei, China; University of Michigan Medical School, Ann Arbor, Michigan, USA

**Keywords:** PRRSV, FcRn, N protein, uncoating

## Abstract

Porcine reproductive and respiratory syndrome virus (PRRSV) continues to cause substantial economic losses to the pig industry worldwide. Previous studies from other groups showed that CD163 is required for PRRSV uncoating and genome release. However, CD163 does not interact with nucleocapsid (N) protein. In this study, the neonatal Fc receptor (FcRn) was demonstrated to be irreplaceable for PRRSV infection by knockdown, overexpression, antibodies or IgG blocking, knockout, and replenishment assays. FcRn was further revealed to be involved in PRRSV uncoating for the first time rather than viral attachment and internalization. In detail, FcRn was determined to colocalize with CD163 and PRRSV virions in early endosomes and specially interact with N protein in early endosomes. Taken together, these results provide evidence that FcRn is an essential cellular factor for PRRSV uncoating, which will be a promising target to interfere with the viral infection.

**IMPORTANCE:**

PRRSV infection results in a severe swine disease affecting pig farming in the world. Although CD163 has been implicated as the uncoating receptor for PRRSV but the uncoating mechanism of PRRSV remains unclear. Here, we identified that FcRn facilitated virion uncoating *via* interaction with viral N protein in early endosomes. Our work actually expands the knowledge of PRRSV infection and provides an attractive therapeutic target for the prevention and control of PRRS.

## INTRODUCTION

Porcine reproductive and respiratory syndrome (PRRS) is a highly contagious disease caused by PRRS virus (PRRSV). Hallmark symptoms of PRRS are mild to severe respiratory disease in infected newborns and growing pigs and reproductive failure in pregnant sows (1). PRRS has caused huge economic losses to the world’s pig industry since it emerged in North America and Europe almost simultaneously in the late 1980s (1–4). PRRSV is an enveloped, non-segmented, single-stranded positive-sense RNA virus classified within the order *Nidovirales*, family *Arteriviridae*, and genus *Betaarteriviru*s (5). The genome of PRRSV is approximately 15 kb in length and contains at least 10 open reading frames (ORFs) (6–9). Approximately three-quarters of the genome is occupied by two ORFs, ORF1a and ORF1b, which encode two long polyproteins (pp1a and pp1ab) that are cleaved into 16 nonstructural proteins (nsps; nsp1*α*, nsp1*β*, nsp2N, nsp2TF, nsp2-nsp6, nsp7*α*, nsp7*β*, and nsp8-nsp12) by viral proteases (10). Eight structural proteins are encoded by the other eight ORFs, including glycoprotein 2a (GP2a), envelope (E) protein, GP3, GP4, ORF5a protein, GP5, matrix (M) protein, and nucleocapsid (N) protein (6, 7, 9).

PRRSV has a highly restricted tropism for monocyte/macrophages, particularly for porcine alveolar macrophages (PAMs) both *in vivo* and *in vitro* (11, 12). The non-porcine cells known to support PRRSV replication are the African green monkey kidney epithelial cell line MA-104 and its derivatives MARC-145 (13). Up to now, at least eight molecules have been identified as PRRSV candidate receptors, including CD163 (14), CD169 (15), Heparan sulfate (HS) (16, 17), Vimentin (18), CD151 (19), CD209 (20), MYH9 (21), and TIM-1/4 (22). Among them, gene knockout experiments confirmed that CD163 is an irreplaceable receptor for PRRSV infection (23). In addition to SRCR1-4, other domains are important for CD163 as the core receptor of PRRSV (24–27). The CD163 is proposed to be responsible for virions uncoating and genome release in early endosomes where PRRSV GPs (2a, 3, 4, 5) were identified to interact with CD163 (28–30). However, CD163 does not interact with PRRSV N protein (30), and there are currently no studies reporting receptors that interact with viral N protein.

The neonatal Fc receptor (FcRn) was first identified in the intestinal epithelial cells of newborn rats (31). FcRn is a MHC class I-like protein composed of a heavy chain (*α* chain encoded by the *FCGRT* gene) and a light chain (*β*2 microglobulin encoded by the *B2M* gene, *β*2m) to exert biological functions (32–35). The rat FCGRT includes three extracellular regions (α1, α2, and α3), a transmembrane region, and a cytoplasmic tail, with a molecular weight of approximately 50 kDa; *β2m* has a molecular weight of approximately 14 kDa (34). Its functional expression has been demonstrated in a diverse array of cell types, including macrophages (36), neutrophils (37), genital tract epithelium cells (38), intestinal epithelial cells (39), vascular endothelial cells (40), renal epithelial cells (41), etc. In addition to the well-known classic function of FcRn as a transport receptor for IgG, studies in recent years have shown that FcRn acts as an uncoating receptor for some human enteric viruses in early endosomes (32, 35, 42, 43). Whether FcRn is involved in PRRSV infection and what role FcRn plays in viral infection are unclear.

In this study, we show that FcRn plays a vital role during PRRSV infection. We also demonstrate that FcRn interacts with PRRSV M, N proteins in early endosomes and mediates viral uncoating and genome release. Our results provide a new perspective on the early infection process of PRRSV and also find new targets for the prevention and control of PRRS.

## RESULTS

### FcRn is important for PRRSV infection

**Fig 1:**
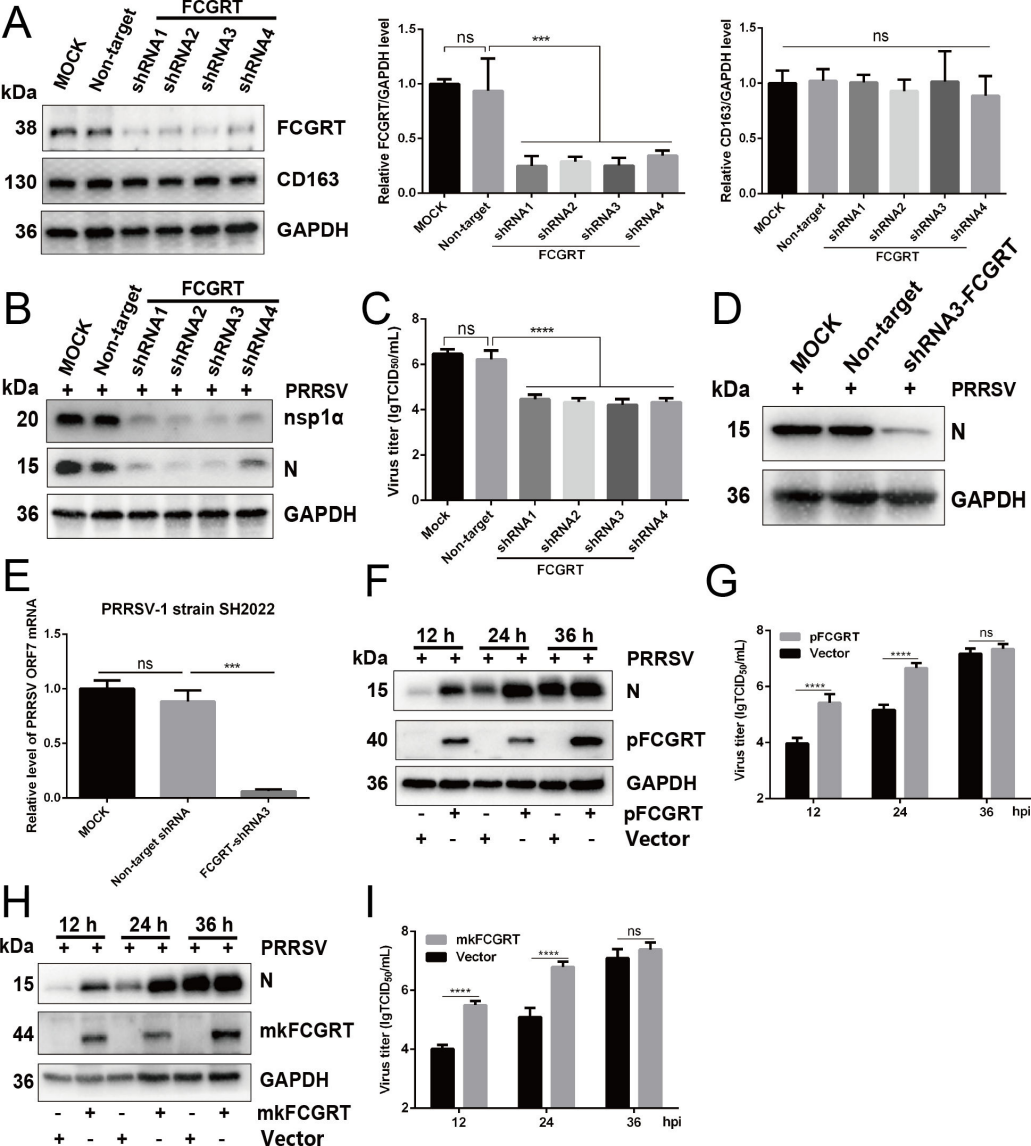
FcRn is important for PRRSV infection in cells. (A–E) *FCGRT* deficiency inhibits PRRSV infection. (**A**) PAMs were transduced with recombinant lentivirus expressing *FCGRT*-specific shRNAs or control lentivirus expressing non-target shRNA. At 36 h after transduction, cells were harvested to determine the knockdown efficiency of *FCGRT* and the CD163 expression level by Western blot using anti-pFCGRT-CT pAb and anti-CD163 mAb. The relative intensity of FCGRT and CD163 protein was quantified by ImageJ software and normalized to GAPDH. (**B and C**) The *FCGRT*-knockdown PAMs or control PAMs were infected with PRRSV strain FJ (MOI of 1.0). At 30 h post-infection (hpi), cells were harvested to determine viral protein expression by Western blot (**B**) with anti-N protein pAb and anti-nsp1*α* pAb or supernatant PRRSV titers by TCID_50_ assay (C). (**D and E**) The *FCGRT*-knockdown PAMs or control cells were infected with PRRSV-2 strain SH (MOI of 1.0) or PRRSV-1 strain SH2022 (MOI of 1.0) for 30 h, respectively. Cells were subjected to Western blot to detect viral N protein expression with anti-N protein pAb (**D**) and qPCR to evaluate the expression of *ORF7* (**E**). (F–I) Overexpression of *FCGRT* promotes PRRSV proliferation. MARC-145 cells were transfected with the pCMV-Tag2B-p*FCGRT* (**F and G**), pCMV-Tag2B-mk*FCGRT* (**H and I**), or empty vector for 36 h, followed by PRRSV strain FJ (MOI of 1.0) infection. At 12, 24, and 36 hpi, cells were collected to determine viral N protein expression by Western blot (**F and H**) with anti-N protein pAb or supernatant PRRSV titers by TCID_50_ assay (**G and I**). Data represent means ± SD from three independent experiments. Significant differences from results with the control group are indicated as follows: ***P < 0.001; ****P < 0.0001; ns, not significant (*P* > 0.05).

Our preliminary experimental results showed that both PAMs and MARC-145 cells express the FcRn protein. To investigate whether FcRn affects PRRSV infection, two strategies were employed: knockdown by specific short hairpin RNA (shRNA) and overexpression. Considering the higher expression levels of FcRn in PAMs, we designed four shRNAs against porcine FCGRT and examined their interference effects in PAMs. We first examined the knockdown efficiency of FCGRT and the effect of knocking down FCGRT on the expression level of CD163 protein by Western blot analysis. As shown in Fig. 1A, the four shRNAs exhibited visible knockdown effects on FCGRT expression and had no obvious effect on CD163 expression compared to the non-target shRNA group. At the same time, non-target shRNA had no distinct effect on FCGRT and CD163 expression compared to the MOCK group. Furthermore, cell viability was assessed using a cell counting kit-8 (CCK-8) assay, which showed that the knockdown of FCGRT had no effect on PAM growth within 72 h (Fig. S1A). Subsequently, The FCGRT-knockdown PAMs or control cells were inoculated with PRRSV-2 sublineage 8.7 strain FJ (multiplicity of infection [MOI]=1) and then assessed by Western blot and 50% tissue culture infected dose (TCID_50_) assay, respectively. The Western blot results were consistent with TCID_50_ data, showing a marked decrease in PRRSV nsp1α, N protein levels, and the virus titers in FCGRT-knockdown PAMs compared to the non-target shRNA group at 30 h post-infection (hpi) (Fig. 1B and C).

PRRSV-2 lineage 1 is currently circulating in China and the United States (44, 45). PRRSV-1 is mainly prevalent in Europe, but in recent years PRRSV-1 has appeared sporadically in many provinces in China (46). To investigate the effect of FCGRT deficiency on NADC30-like PRRSV (PRRSV-2 lineage 1) or PRRSV-1 infection, shRNA3 was selected for subsequent experiments. The FCGRT-knockdown PAMs or control cells were inoculated with PRRSV-2 strain SH (Sublineage 1.8) at 1 MOI or PRRSV-1 strain SH2022 (MOI = 1). Western blot analysis revealed that FCGRT deficiency dramatically reduced the viral N protein level compared to the non-target shRNA group at 30 hpi (Fig. 1D), and qPCR results also showed that knocking down FCGRT significantly diminished the expression of PRRSV-1 mRNA (Fig. 1E), indicating that FcRn deficiency remarkably inhibits PRRSV infection in PAMs.

The aforementioned results verified the positive regulatory role of FcRn in PRRSV infection. To further confirm the role, we explored the effect of overexpression of FCGRT on PRRSV infection. MARC-145 cells were transfected with the porcine (*P*) or green monkey (mk) FCGRT expression constructs or empty vector for 36 h followed by PRRSV infection for indicated times. As expected, the results showed that the PRRSV N protein expression (Fig. 1F and H) and virus titers (Fig. 1G and I) at 12 and 24 hpi were significantly elevated by FCGRT overexpression, suggesting that FcRn indeed has a positive regulatory effect on PRRSV proliferation. Based on the above-described results from knockdown and overexpression assays, we concluded that FcRn is crucial for PRRSV infection.

### FcRn antibodies and IgG block PRRSV infection in cells

**Fig 2:**
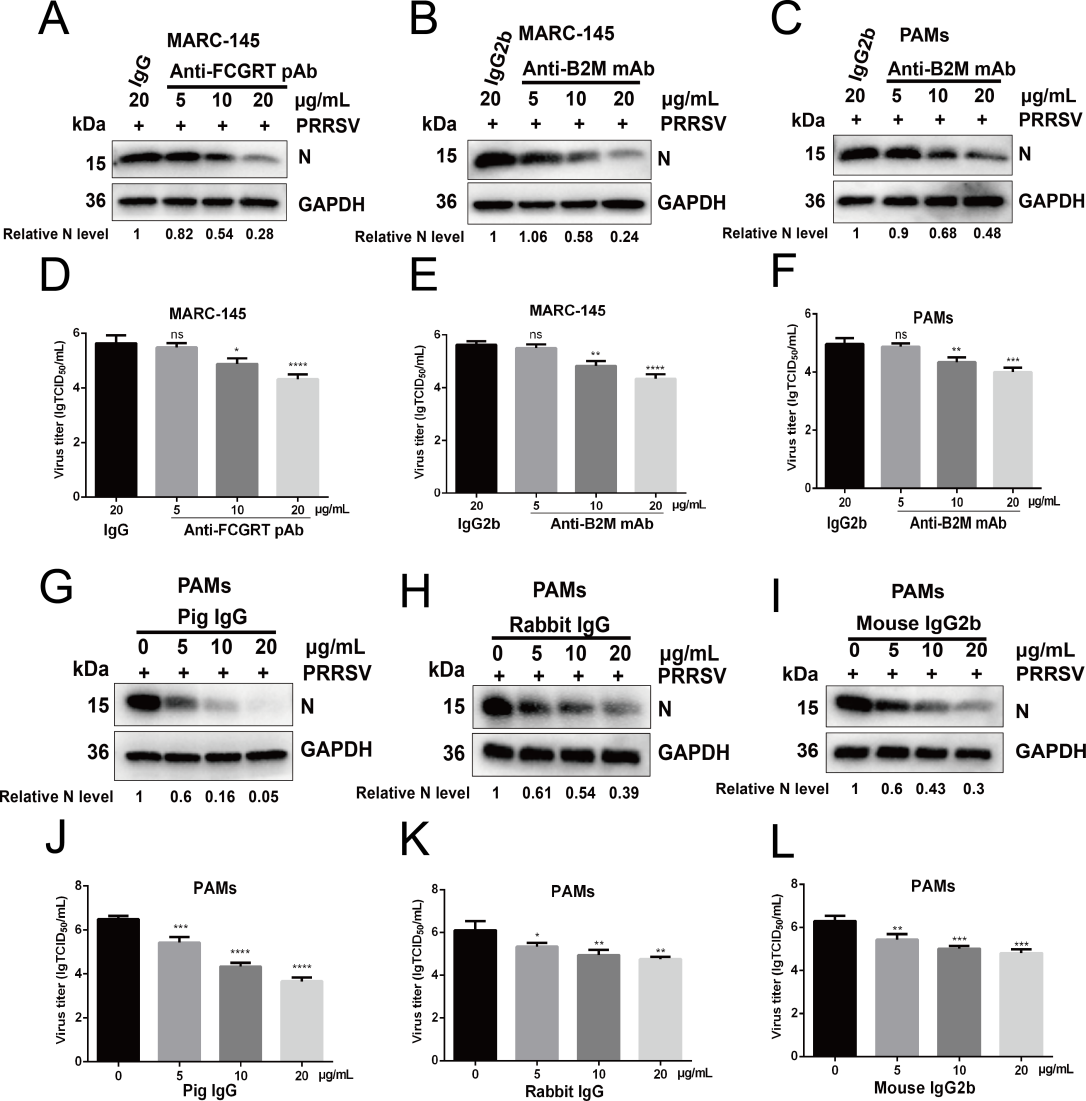
FcRn antibodies and IgG block PRRSV infection. (A–F) FcRn antibodies block PRRSV infection. (A, B, D, and E) MARC-145 cells were incubated with indicated antibodies or isotype IgG at the indicated concentrations (5, 10, and 20 µg/mL) for 1 h at 37°C prior to PRRSV strain FJ (MOI of 1.0) infection. The infected cells were cultured in the presence of antibodies or isotype IgG and harvested at 30 hpi to determine viral N protein expression by Western blot (**A and B**) with anti-N protein pAb or supernatant PRRSV titers by TCID_50_ assay (**D and E**). (**C and F**) PAMs were pretreated with B2M mAb or isotype IgG at the indicated concentrations for 1 h at 37°C. After inoculation with PRRSV strain FJ (MOI of 1.0), the infected cells were cultured in the presence of antibody or IgG2b and harvested at 30 hpi to determine viral N protein expression by Western blot (**C**) with anti-N protein pAb or supernatant PRRSV titers by TCID_50_ assay (**F**). (G to L) IgG blocks PRRSV infection. Prior to PRRSV strain FJ (MOI of 1.0) infection, PAMs were pretreated with IgG from different species (pig, rabbit, and mouse) at the indicated concentrations for 1 h at 37°C. The infected cells were cultured in the presence of IgG and then collected at 30 hpi to determine viral N protein expression by Western blot (G–I) with anti-N protein pAb or supernatant PRRSV titers by TCID_50_ assay (J**–**L). ImageJ software was used to analyze the relative levels of PRRSV N protein in comparison with a control group, and the ratios are displayed as fold changes below the images. Data represent means ± SD from three independent experiments. Significant differences from results with the control group are indicated as follows: *P < 0.05; **P < 0.01; ***P < 0.001; ****P < 0.0001; ns, not significant (*P* > 0.05).

To explore the possibility that FcRn serves as a key host factor during PRRSV infection, we tested whether FcRn antibodies could block PRRSV infection *in vitro*. First, we tested the toxicity of FcRn antibodies in MARC-145 cells and PAMs, and the CCK-8 results confirmed that cell viability was unaffected by FcRn antibodies at the highest concentration (40 µg/mL) (Fig. S2A through C). Next, MARC-145 cells or PAMs were pretreated with antibodies or isotype IgG for 1 h at 37°C prior to PRRSV infection. As shown in Fig. 2A and B, Western blot analysis showed that both FCGRT polyclonal antibody (pAb) and B2M monoclonal antibody (mAb) inhibit the PRRSV N protein expression in MARC-145 cells at 30 hpi, indicating that PRRSV infection could be inhibited by FcRn antibodies. The B2M mAb showed a similar inhibitory effect on PRRSV infection of PAMs (Fig. 2C). These phenotypes were also confirmed by TCID_50_ assay, in which PRRSV titers in cells treated with FcRn antibodies were significantly lower than that in IgG control cells (Fig. 2D through F). The inhibitory effects started at 10 µg/mL and raised with increasing amounts of antibodies compared to control IgG. These results are indicative that FcRn may be required for PRRSV infection.

FcRn is known to bind IgG. We wanted to know whether FcRn binding to IgG affects PRRSV infection. We also first examined the toxicity of pig and rabbit IgG to PAMs, and the CCK-8 results showed that cell viability was unaffected by the IgG (Fig. S2D and E). Subsequently, PAMs were treated with IgG and then inoculated with PRRSV. In Fig. 2G through I, Western blot results showed that IgG from different species has dose-dependent inhibitory effects on PRRSV N protein expression in PAMs compared to the control group at 30 hpi. Similar results were observed in Fig. 2J through L by assessing TCID_50_. It is worth mentioning that 20 µg/mL swine IgG displayed the most remarkable suppression effect on PRRSV infection in PAMs (Fig. 2G and J). However, PRRSV infection could not be inhibited by 40 µg/mL rabbit or mouse IgG in MARC-145 cells (Fig. S2F and G). Taking these results together, we showed that FcRn may be a critical host factor for PRRSV to enter cells.

### Loss of FcRn expression renders MARC-145 cells resistant to PRRSV infection

**Fig 3:**
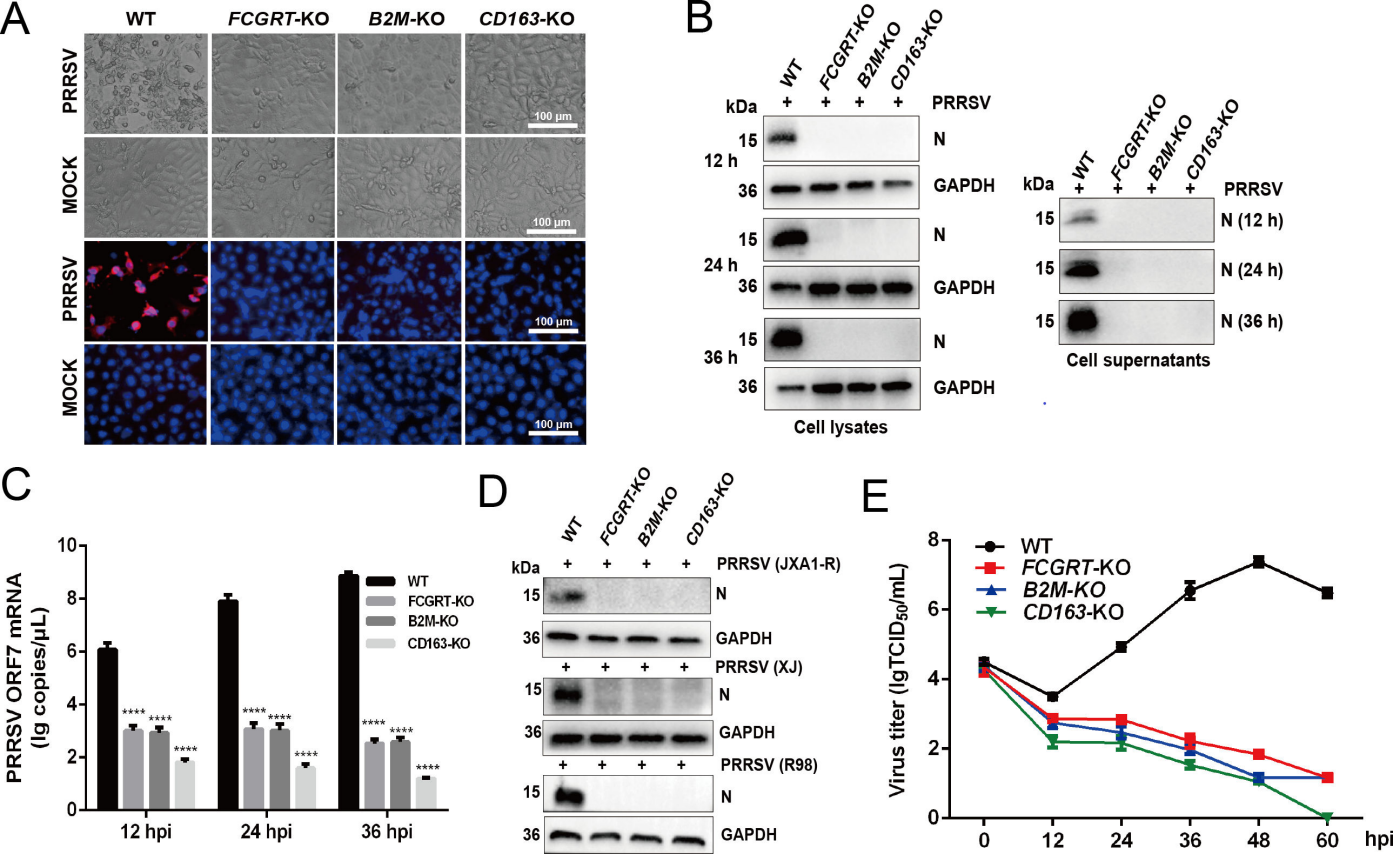
Loss of FcRn expression renders MARC-145 cells resistant to PRRSV infection. (**A**) Light microscopy images showing CPE of *FCGRT*-KO, *B2M*-KO, *CD163*-KO, and WT MARC-145 cells at 48 hpi with PRRSV-2 strain FJ (MOI of 1.0) (upper two panels). Cells were MOCK-infected or infected with PRRSV-2 strain FJ (MOI of 1.0) for 48 h, and IFA was performed to detect the viral fluorescence signals with anti-N protein pAb (red) (lower two panels). Nuclei were stained with DAPI (blue). Scale bars represent 100 µm, and representative images are shown. (**B and C**) The three single-gene KO and WT MARC-145 cells were infected with PRRSV-2 strain FJ (MOI of 1.0) for 12, 24, and 36 h. PRRSV-infected cells were collected to detect viral N protein expression by Western blot (**B**) with anti-N protein pAb or PRRSV ORF7 mRNA expression by qPCR (**C**). (**D**) The three single-gene KO and WT MARC-145 cells were inoculated with PRRSV-2 strain JXA1-R (MOI of 1.0), XJ (MOI of 1.0), and R98 (MOI of 1.0) for 30 h, and then the infected cells were harvested to detect the viral N protein expression by Western blot with anti-N protein pAb. (**E**) Growth curve of PRRSV in *FCGRT*-KO, *B2M*-KO, *CD163*-KO, and WT MARC-145 cells. Cells were inoculated with PRRSV strain FJ (MOI 0.1), and then the inoculums were removed after 1 h. After twice washes with PBS, the cells were added with the fresh media containing 2% FBS, and the supernatants were collected at 12, 24, 36, 48, and 60 hpi, respectively, and then the virus titers at indicated time points were calculated by TCID_50_ assay. Data represent means ± SD from three independent experiments. Significant differences from results with the control group are indicated as follows: ****P < 0.0001.

We next determined whether loss of FcRn expression renders MARC-145 cells less susceptible to infection. For the study, we utilized CRISPR/Cas9-mediated gene knockout (KO) technology to construct three single-gene (*FCGRT*, *B2M*, and *CD163*) KO MARC-145 cell lines (Fig. S3A through E). Meanwhile, the KO of three single-gene had no significant effect on cell viability compared to the wild-type (WT) cells (Fig. S3F). Subsequently, *FCGRT*-KO, *B2M*-KO, *CD163*-KO, and WT cells were inoculated with PRRSV. Cytopathic effect (CPE) was undetectable in *FCGRT*-KO, *B2M*-KO, and *CD163*-KO cells at 48 hpi (Fig. 3A, upper two panels), and no PRRSV-positive signals (red) were observed in three single-gene KO cells at 48 hpi by immunofluorescence staining (Fig. 3A, lower two panels). Western blot results showed that no expression of PRRSV N protein was observed in three single-gene KO cell lysates and supernatants at 12, 24, and 36 hpi (Fig. 3B), and qPCR data demonstrated that the viral ORF7 copies in three single-gene KO cells was significantly reduced compared with that in WT cells at 12, 24, and 36 hpi (Fig. 3C). To rule out the contingency of these results, multiple *FCGRT*-KO and *B2M*-KO monoclonal cells were inoculated with PRRSV. As shown in Fig. S3G, the PRRSV N protein expression in all monoclonal cells was undetectable by Western blot at 30 hpi. To further confirm that FcRn is important for multiple PRRSV strains infection, three single-gene KO and WT cells were inoculated with PRRSV-2 strain XJ (sublineage 8.7), JXA1-R (attenuated vaccine strain from HP-RRSV strain JXA1), and R98 (sublineage 5.1). As shown in Fig. 3D, the viral N protein expression of three PRRSV strains in three single-gene KO cells was also not detected by Western blot at 30 hpi, consistent with the result of PRRSV-2 strain FJ. Furthermore, TCID_50_ data demonstrated that no obvious viral proliferation was observed in the three single-gene KO cells spanning 60 h (Fig. 3E). Collectively, these results supported that FcRn expression is required for PRRSV proliferation.

### Replenishment of FcRn in FcRn-KO cells restores PRRSV proliferation

**Fig 4:**
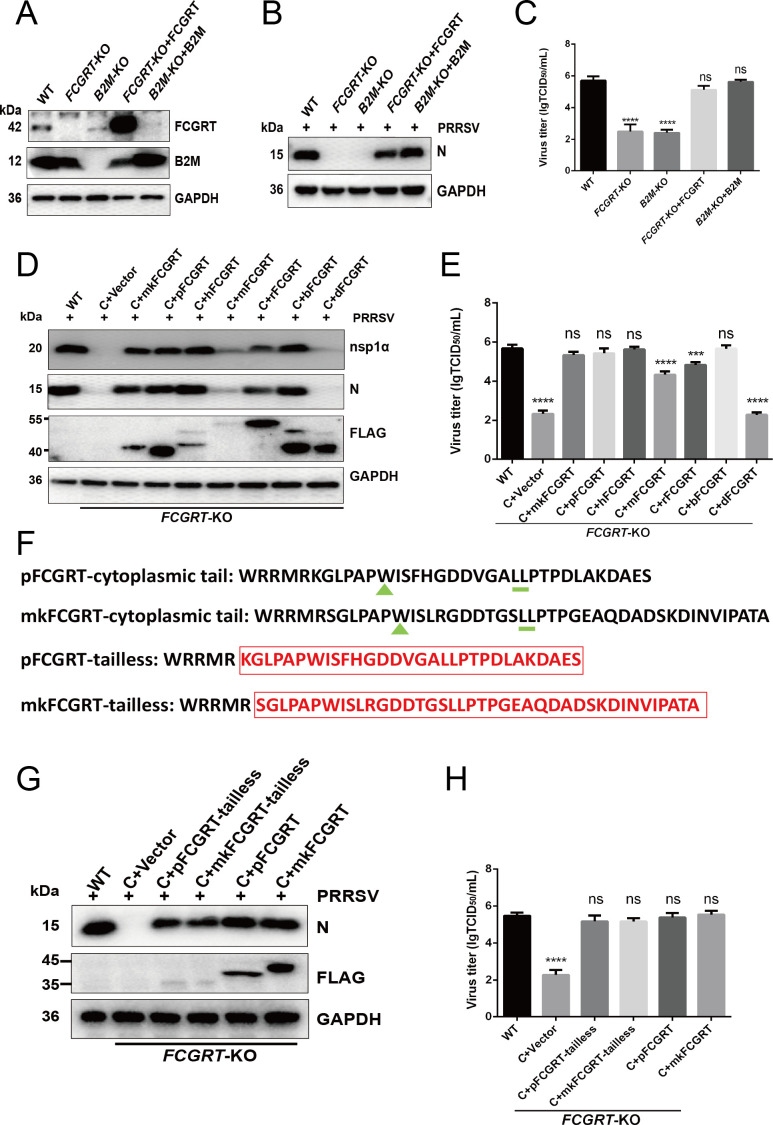
The replenishment of FcRn in FcRn-KO MARC-145 cells recovers PRRSV infection (A–C) Replenishing green monkey FcRn into FcRn-KO cells restores PRRSV proliferation. (**A**) The *FCGRT*-KO and *B2M*-KO cells were transduced with recombinant lentivirus expressing mkFCGRT and mkB2M for 48 h, respectively, and then the cells were dealt with 1 mg/mL hygromycin B for 72 h. Subsequently, the expression of mkFCGRT and mkB2M was identified by Western blot with anti-FCGRT pAb and anti-B2M mAb. (**B and C**) The *FCGRT*-KO + mkFCGRT and *B2M*-KO + mkB2 M cells were infected with PRRSV strain FJ (MOI of 1.0) for 30 h, and then infected cells were harvested to determine the viral N protein expression by Western blot (**B**) with anti-N protein pAb or PRRSV titers by TCID_50_ assay (**C**). (**D and E**) The *FCGRT*-KO cells were transduced with recombinant lentivirus expressing FCGRT from different species (green monkey, pig, human, mouse, rat, bovine, and dog) or empty vector lentivirus for 48 h, and then these cells were treated with 1 mg/mL hygromycin B for 72 h. After inoculation with PRRSV strain FJ (MOI of 1.0) for 30 h, these infected cells were collected to detect the expression of viral N protein, nsp1*α,* and FCGRT-FLAG fusion protein by Western blot (**D**) with anti-N protein pAb, anti-nsp1*α* pAb*,* and anti-FLAG mAb or PRRSV titers by TCID_50_ assay (**E**). (**F**) Amino acid sequences of pig and green monkey FCGRT cytoplasmic tail. The green triangles indicate the tryptophan residue, the green short lines indicate the dileucine residue, and the red boxes indicate the deleted amino acid sequence of the FCGRT cytoplasmic tail. (**G and H**) The *FCGRT*-KO cells were transduced with recombinant lentivirus expressing pFCGRT-tailless and mkFCGRT-tailless or empty vector lentivirus for 48 h, and then cells were dealt with Hygromycin B (1 mg/mL) for 72 h. The *FCGRT*-KO +pFCGRT-tailless, *FCGRT*-KO +mkFCGRT-tailless, and control cells were infected with PRRSV strain FJ (MOI of 1.0) for 30 h, and then cells were harvested to detect the expression of viral N protein and FCGRT-tailless-FLAG fusion protein by Western blot (**G**) with anti-N protein pAb and anti-FLAG mAb or PRRSV titers by TCID_50_ assay (**H**). Data represent means ± SD from three independent experiments. Significant differences from results with the control group are indicated as follows: ***P < 0.001; ****P < 0.0001; ns, not significant (*P* > 0.05).

To further confirm that FcRn is required for PRRSV proliferation, green monkey FCGRT and B2M were introduced into *FCGRT*-KO and *B2M*-KO cells, respectively (Fig. 4A). Subsequently, *FCGRT-*KO + FCGRT and *B2M*-KO + B2 M cells were inoculated with PRRSV. As shown in Fig. 4B and C, Western blot and TCID_50_ results showed that the complementation of FCGRT and B2M into *FCGRT*-KO and *B2M*-KO cells, respectively, restores PRRSV proliferation. FcRn is conserved in different mammals (Fig. S4A). We want to explore the impact of FCGRT from different species on PRRSV proliferation. After pig, human, mouse, rat, bovine, and dog FCGRT were introduced into *FCGRT*-KO cells, these cells were inoculated with PRRSV. Western blot and TCID_50_ results showed that FCGRT from pigs, green monkeys, humans, and bovines could completely restore PRRSV proliferation, and FCGRT from mice or rats could partially restore PRRSV proliferation, while FCGRT from dogs could not recover PRRSV proliferation (Fig. 4D and E). These results further confirmed that FcRn is indeed involved in PRRSV proliferation.

The dileucine residues and tryptophan residues in the FCGRT cytoplasmic tail are critical for FcRn-mediated endocytosis and intracellular trafficking (47). We constructed tailless pFCGRT and mkFCGRT plasmids (Fig. 4F), leaving five residues (WRRMR) allowing proper insertion of FcRn into the membrane (48, 49). To investigate whether the proliferation of PRRSV in MARC-145 cells depends on the FcRn cytoplasmic tail, green monkey and pig FCGRT-tailless were introduced into *FCGRT*-KO cells, respectively, followed by inoculation with PRRSV. Western blot and TCID_50_ results, pictured in Fig. 4G and H, showed that green monkey and porcine FCGRT-tailless recover PRRSV proliferation, although the level of viral replication is slightly lower compared to WT FCGRT. These results illustrated that PRRSV proliferation in MARC-145 cells is independent of the FcRn cytoplasmic tail.

### PRRSV virions are arrested before the formation of RTCs in FcRn-KO MARC-145 cells

**Fig 5:**
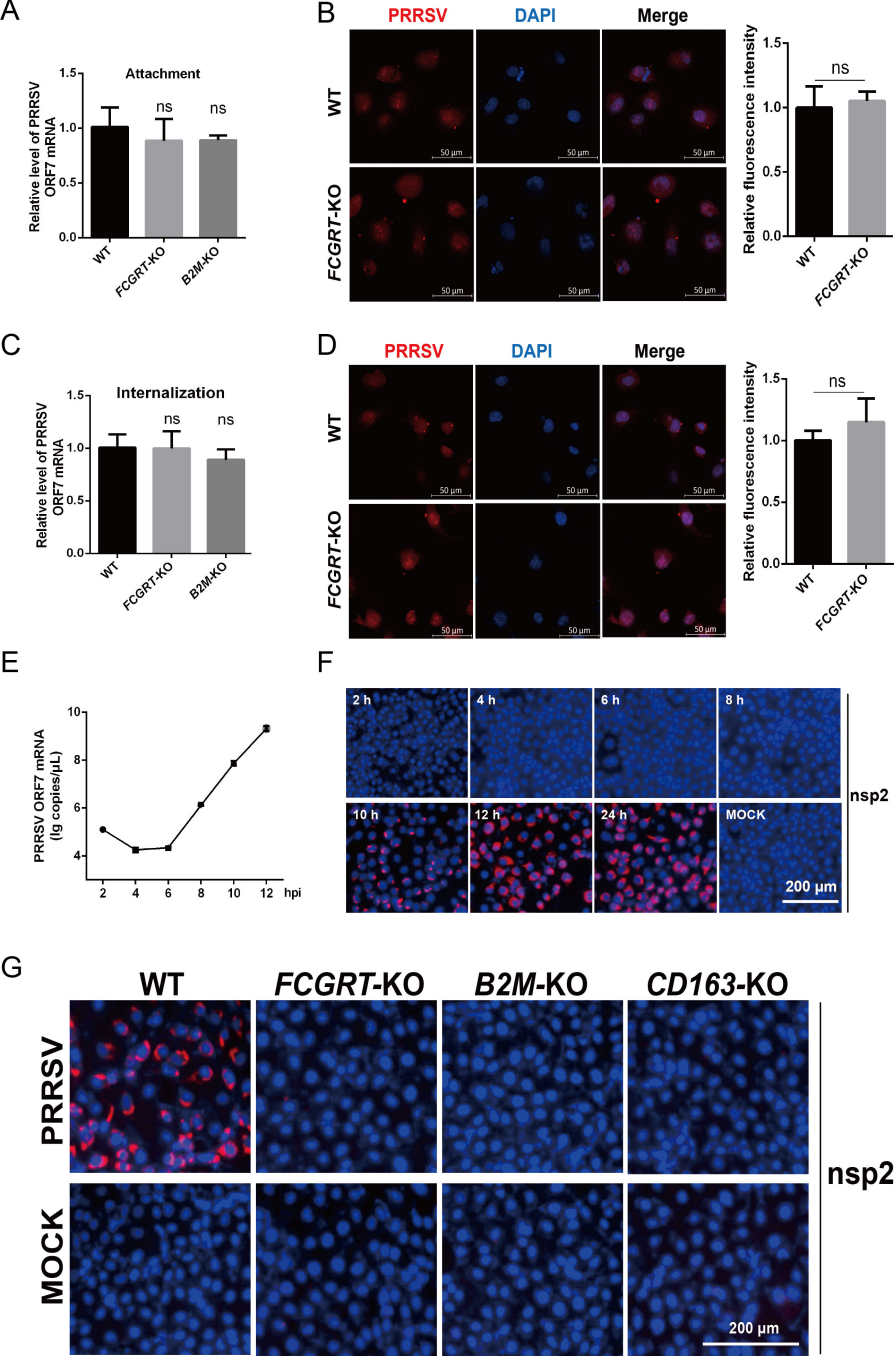
The arrest in infection of FcRn-KO MARC-145 cells occurs prior to the formation of the replication transcription complexes (RTCs). (**A and B**) Adsorption assay. The FcRn-KO and WT MARC-145 cells cultured in six-well plates or confocal dishes were prechilled at 4°C for 1 h, and then media were replaced by precooled DMEM containing PRRSV strain FJ (MOI of 1.0). After incubation at 4°C for 2 h, the cells were washed with precooled PBS and harvested to determine PRRSV RNA abundance using qPCR (**A**) or viral N protein fluorescence density using confocal microscopy (**B**). Nuclei were stained with DAPI (blue). Scale bars represent 50 µm, and representative images are shown. The total fluorescence intensity of the N protein was calculated using ImageJ software. (**C and D**) Penetration assay. The FcRn-KO and WT MARC-145 cells cultured in six-well plates or confocal dishes were prechilled at 4°C for 1 h and then incubated with PRRSV (MOI of 1.0) at 4°C for 2 h. The virus-containing media was replaced by fresh media containing 2% FBS, and the temperature was shifted to 37°C for 2 h. After washing with PBS, the cells were collected to determine PRRSV RNA abundance using qPCR (**C**) or viral N protein fluorescence density using confocal microscopy (**D**). Nuclei were stained with DAPI (blue). Scale bars represent 50 µm, and representative images are shown. The total fluorescence intensity of N protein was calculated using ImageJ software. (**E**) MARC-145 cells grown in 24-well plates were infected with PRRSV strain FJ (MOI of 1.0) at 37°C for 1 h, and then cells were washed thoroughly with PBS to remove uninternalized virions. The infected cells were cultured with fresh media for the indicated time, and then cells were collected every 2 h until at 12 hpi to determine the intracellular PRRSV RNA abundance using qPCR. (**F**) MARC-145 cells were treated as described for panel E. The cells supernatants in each well were harvested every 2 h from 2 hpi to 12 hpi or 24 hpi, and fresh MARC-145 cells were inoculated with harvested supernatants for 36 h. IFA was performed to detect the viral fluorescence signals with mAb against PRRSV nsp2 (red). Nuclei were stained with DAPI (blue). The scale bar represents 200 *µ*m, and representative images are shown. (**G**) The *FCGRT*-KO, *B2M*-KO, *CD163*-KO, and WT MARC-145 cells were MOCK-inoculated (bottom) or inoculated (upper) with PRRSV strain FJ (MOI of 1.0) for 24 h, and then the infected cells were fixed for immunofluorescent staining of PRRSV nsp2 (red). Nuclei were stained with DAPI (blue). The scale bar represents 200 µm, and representative images are shown. Data represent means ± SD from three independent experiments. significant differences from results with the control group are indicated as follows: ns, not significant (*P* > 0.05).

The life cycle of PRRSV can be divided into attachment, internalization, uncoating, replication, translation, assembly, and release (10). To assess the involvement of FcRn in the PRRSV life cycle, we first examined the effect of FcRn-KO on viral attachment. FcRn-KO and WT MARC-145 cells were inoculated with PRRSV, and then an adsorption assay was performed. As shown in Fig. 5A and B, there are no significant differences in PRRSV RNA abundance and N protein fluorescence intensity between FcRn-KO and WT cells, suggesting that FcRn is not involved in PRRSV adsorption to MARC-145 cells. We subsequently inspected whether FcRn played a role in PRRSV internalization. The results, depicted in Fig. 5C and D, displayed that viral RNA abundance and N protein fluorescence intensity were no significant differences between FcRn-KO and WT cells, implying that FcRn is not necessary for PRRSV internalization into MARC-145 cells.

Next, we delineated the kinetics of PRRSV RNA replication and determined the time point at which infectious progeny viruses were produced in MARC-145 cells. PRRSV RNA copies began to rise at six hpi (Fig. 5E), and infectious progeny viruses were released into the supernatants at 10 hpi (Fig. 5F). The results pointed out that the life cycle of PRRSV strain FJ is 10 h.

As the PRRSV genome is released into the cytoplasm, they initiate the translation process, and subsequently, some viral nsps make up replication and transcription complexes (RTCs) (10). The RTCs existing in the cells represent the initiation of viral replication, and nsp2, involved in the formation of double-membrane vesicles, was often chosen as a representative marker for the RTCs (10, 24, 30). The absence of replicated PRRSV ORF7 gene and lack of expression of N protein in the PRRSV-infected FcRn-KO cells (Fig. 3) might be the fact that the PRRSV virions are arrested in endosomes before the formation of RTCs. To verify this hypothesis, *FCGRT*-KO, *B2M*-KO, *CD163*-KO, and WT MARC-145 cells were inoculated with PRRSV for 24 h, and then cells were stained for PRRSV nsp2 following permeabilization. We found that RTCs were presented in WT cells but not in three single-gene KO cells (Fig. 5G), consistent with previous reports (24, 30), implying that FcRn may be involved in PRRSV uncoating and genome release. Together, these results verified that PRRSV failed to initiate productive infection, and the infection was arrested before the formation of RTCs in FcRn-KO MARC-145 cells.

### FcRn interacts with PRRSV in early endosomes during early entry

**Fig 6:**
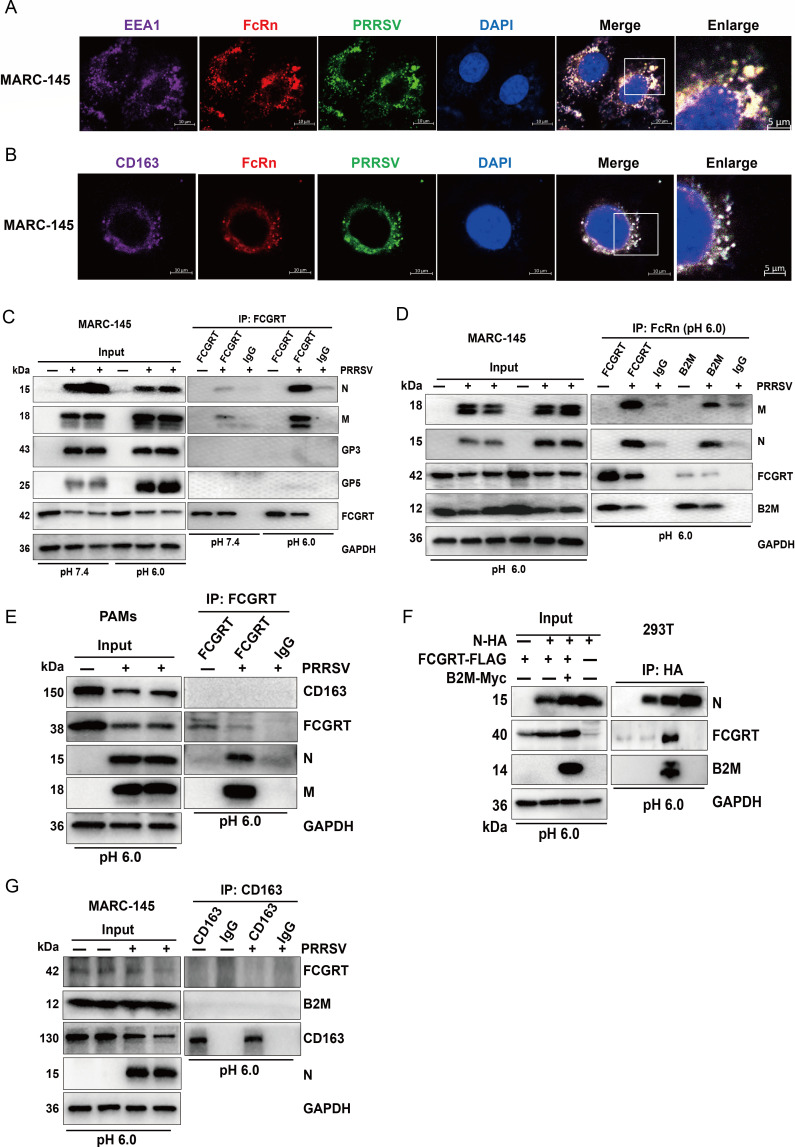
FcRn interacts with PRRSV M and N proteins in early endosomes. (**A**) MARC-145 cells seeded in a confocal dish were prechilled at 4°C for 1 h, and then the media were replaced by precooled DMEM containing PRRSVstrain FJ (MOI of 1.0). After incubation at 37°C for 30 min, the cells were first costained with mouse anti-EEA1 mAb (purple), rabbit anti-FCGRT pAb (red), and pig anti-PRRSV pAb (green), respectively. After washes with PBS, the cells were costained with CoraLite647-conjugated goat anti-mouse IgG and ABflo594-conjugated goat anti-rabbit IgG. After another wash with PBS, the cells were finally stained with FITC-conjugated rabbit anti-pig IgG and then detected using confocal microscopy. Nuclei were stained with DAPI (blue). Scale bars represent 10 µm, and representative colocalization (white) images are shown. (**B**) MARC-145 cells were dealt with as described for panel A, but the primary antibody (EEA1 mAb) was replaced with mouse mAb against CD163. Scale bars represent 10 µm, and representative colocalization (white) images are shown. (**C**) *FCGRT*-KO + mkFCGRT-FLAG MARC-145 cells cultured in six-well plates were incubated with PRRSV strain FJ (MOI of 5.0) for 0.5 h at 37°C. After washes with PBS, the cells were lysed with RIPA (pH 7.4 or 6.0) and immunoprecipitated with anti-FLAG mAb. The whole-cell lysates (input) and immunoprecipitation (IP) complexes were analyzed by Western blot with anti-FCGRT, anti-B2M, anti-PRRSV (**N, M, GP5, GP3**), and anti-GAPDH antibodies, respectively. (**D**) *FCGRT*-KO +mkFCGRT-FLAG MARC-145 cells were dealt with as described for panel C. The cells were lysed with RIPA (pH 6.0) and immunoprecipitated with anti-FLAG mAb or B2M mAb. The input and IP complexes were analyzed by Western blot with anti-FCGRT, anti-B2M, anti-PRRSV N protein, anti-PRRSV M protein, and anti-GAPDH antibodies, respectively. (**E**) PAMs were treated as described for panel C. The cells were lysed with RIPA (pH 6.0) and immunoprecipitated with rabbit anti-pFCGRT-CT pAb. The input and IP complexes were analyzed by Western blot with anti-pFCGRT-CT, anti-N protein, anti-M protein, anti-CD163, and anti-GAPDH antibodies, respectively. (**F**) HEK-293T cells were cotransfected with expression vectors encoding HA-tagged PRRSV N protein, FLAG-tagged pFCGRT, and Myc-tagged pB2M. The cells were lysed at 48 h post-transfection and then immunoprecipitated with anti-HA antibody. The input and IP complexes were analyzed by western blot with anti-FLAG, anti-HA, anti-Myc, and anti-GAPDH antibodies, respectively. (**G**) MARC-145 cells stably expressing mkCD163-HA were incubated with PRRSV strain FJ (MOI of 1.0) for 24 h. The infected cells were lysed with RIPA (pH 6.0) and then immunoprecipitated with anti-HA antibody. The input and IP complexes were analyzed by western blot with anti-FCGRT, anti-B2M, anti-CD163, anti-PRRSV N protein, and anti-GAPDH antibodies, respectively.

Regardless of PAMs or MARC-145 cells, PRRSV virions enter CD163-positive early endosomes after internalization (29, 30), and the interactions between CD163 and GPs (2a, 3, 4, 5) may mediate PRRSV uncoating and genome release (28, 30, 50). The nucleocapsid (N) proteins wrap the PRRSV genomic RNA to form a core structure, which plays a significant role in protecting the viral nucleic acid (51). However, CD163 does not interact with N protein (30). Therefore, we wanted to explore whether FcRn mediates PRRSV uncoating and genome release by interacting with PRRSV N protein in early endosomes. After 30 min of viral internalization, we first carried out confocal microscopy to examine the localization of FcRn, PRRSV, and EEA1 (served as the marker protein of early endosomes) (29, 30). As shown in Fig. 6A, the colocalization of FcRn with virions and EEA1 was observed in MARC-145 cells. Under the same conditions, we then explored the localization of FcRn, CD163, and virions. Confocal microscopy results showed that FcRn colocalizes with CD163, and virions (Fig. 6B). The same results were also observed in PAMs (Fig. S5A and B). These results indicate that PRRSV virions will enter CD163-positive FcRn-positive early endosomes after internalization.

To further investigate whether FcRn interacts with PRRSV in early endosomes, *FCGRT*-KO + mkFCGRT MARC-145 cells were infected with PRRSV (MOI = 5), and Co-immunoprecipitation assay was conducted. The results revealed that FcRn interacts with PRRSV M and N proteins at 0.5 hpi, but not GP3 and GP5, and the interactions were much stronger at pH 6.0 than at pH 7.4 (Fig. 6C and D). The interactions between FcRn and viral M and N proteins could also be detected in PRRSV-infected PAMs (Fig. 6E). Since the anti-N protein antibody cannot be used for immunoprecipitation, the eukaryotic plasmids expressing pFCGRT, pB2M, and PRRSV N protein were cotransfected into HEK-293T cells for 48 h, and the interaction between FcRn and N protein was further confirmed by immunoprecipitating the N-HA fusion protein using anti-HA antibody (Fig. 6F). Because PRRSV virions enter CD163-positive FcRn-positive early endosomes after internalization (Fig. 6A and B, Fig. S5A and B), we wanted to know whether there is an interaction between FcRn and CD163. As shown in Fig. 6E and G, regardless of the presence or absence of PRRSV infection, FcRn does not interact with CD163. Collectively, these results confirmed that FcRn interacts with PRRSV N, M proteins in early endosomes.

## DISCUSSION

During the entire process of virus entry, the complex virus-host interaction network is essential, and viral structural proteins interact with host receptors to mediate viral invasion. Although cells and animal experiments have confirmed that CD163 is an indispensable receptor for PRRSV infection (14, 23) and suggested its crucial role in viral uncoating and genome release (24, 30, 52), no studies have conclusively identified CD163 as the uncoating receptor of PRRSV. In this study, FcRn was found to interact with PRRSV N and M proteins and played a vital role in viral uncoating and genome release.

The knockdown and overexpression experiments confirmed that FcRn has a positive regulatory effect on PRRSV infection (Fig. 1). Knocking out either *FCGRT* or *B2M*, which encode the two subunits of FcRn, confers MARC-145 cells resistant to PRRSV infection (Fig. 3), indicating that viral proliferation requires complete FcRn molecule. This is similar to the phenomenon that knocking out FcRn renders HEK-293T cells resistant to echovirus infection (32, 35). Overexpression of either porcine FCGRT or green monkey FCGRT enhanced PRRSV proliferation in MARC-145 cells at 12 and 24 hpi (Fig. 1F through I), suggesting that porcine FCGRT can also form a chimeric FcRn with a green monkey (mk) B2M (Fig. S4B). The overexpression of FcRn did not significantly enhance PRRSV proliferation at 36 h, possibly because the viral load of the cells reached its peak. The high homology of FCGRT in different species also provides the possibility for the formation of chimeric FcRn (Fig. S4A). The results showed that the complement of FCGRT from mice or rats in *FCGRT*-KO MARC-145 cells could partially restore PRRSV proliferation while replenishing canine FCGRT could not recover the viral proliferation (Fig. 4D and E). It may be because the chimeric FcRn formed by combining mouse or rat FCGRT with mkB2M were different from the mkFcRn in the key amino acid sites required for virus infection, thus resulting in the chimeric FcRn being unable to be effectively utilized by PRRSV. Even if the FCGRT amino acid similarities between dogs and green monkeys are higher than that between mice, rats, pigs, bovine, and green monkeys (Fig. S4A), it is possible that the pivotal amino acids at the junction between canine FCGRT and mkB2M have changed, leading to the unsuccessful formation of chimeric FcRn and subsequent failure of PRRSV infection. Soon afterward, we were surprised to find that PRRSV proliferation does not depend on FcRn cytoplasmic tail (Fig. 4G and H), and after internalization, PRRSV virions enter FcRn-positive early endosomes (Fig. 6A; Fig. S5A). Ye et al. reported that FcRn without the cytoplasmic tail was unable to traffic to early endosomes, but CD74 could restore the localization of tailless FcRn (53), which might explain why FcRn-tailless could also recover PRRSV proliferation. A much lower expression level of tailless FCGRT than full-length FCGRT was also simultaneously observed (Fig. 4G), which might be the shorter half-life of tailless FcRn (54). The CD163 cytoplasmic tail is also not obligatory for PRRSV infection, and whether CD163 is expressed on the cell surface is not related to productive viral infection (27, 55), but its transmembrane region is indispensable for viral replication (14, 28). Consequently, we are inclined to think that productive viral infection only requires membranous FcRn and CD163 in early endosomes, although these facts that the CD163 cytoplasmic tail endocytic motif is necessary for the endocytosis of the hemoglobin-haptoglobin complex (56), and CD163 normally circulates between the cell surface and early endosomes (57), and the FcRn cytoplasmic tail is crucial for FcRn-mediated endocytosis and intracellular trafficking (47). Further research should be undertaken to clarify these issues that the localization of tailless FcRn and CD74 in early endosomes of PRRSV-infected cells, and whether the FcRn transmembrane region is required for PRRSV infection.

FcRn is expressed on both the cell surface and the cytoplasm (35), and the expression in the cytoplasm mainly located in early endosomes (53). Therefore, we conducted antibodies blocking experiments and found that both FCGRT pAb and B2M mAb could effectively block PRRSV infection (Fig. 2A through F), suggesting that the steric hindrance caused by the binding of antibodies to FcRn affects the utilization of FcRn by PRRSV. At the same time, we found that IgG could not block PRRSV infection of MARC-145 cells (Fig. S2F and G), but IgG effectively blocked PRRSV infection of PAMs (Fig. 2G through L). The phenomenon might be due to differences in subcellular localization, expression levels, or physiological functions of FcRn in the two type cells. We found that PAMs have higher FcRn expression levels than MARC-145 cells in our researches (data not shown), and other teams reported that the maintenance of serum IgG and albumin levels relies on the circulating transport function of FcRn in marrow-derived cells (such as macrophages, dendritic cells [58, 59]) and vascular endothelial cells (40). These cells first take up IgG and albumin through pinocytosis, and then FcRn binds IgG or albumin and transports it to the cell surface for release (60). Considering the capability of macrophages to maintain serum homeostasis, we tend to consider that FcRn in PAMs is involved in the cyclic transport of IgG or albumin, thereby preventing PRRSV from taking advantage of FcRn. The disabilities of IgG to block PRRSV infection of MARC-145 cells might be the excessive IgG and albumin in the culture media (FBS) that makes the FcRn of MARC-145 cells in both the control group and experimental group in a saturated binding state, which resulted in the inabilities of added mouse or rabbit IgG to block infection. Swine IgG has a better blocking effect than rabbit and mouse IgG (Fig. 2G through L), which might be the higher affinity of pFcRn to porcine IgG. The above results proved that FcRn, like CD163, is an indispensable factor for PRRSV infection.

We further investigated the specific steps of PRRSV infection in which FcRn plays a role. Previous study verified that FcRn is an adsorption receptor for echovirus 18 (32), while our study showed that knocking out FcRn does not affect PRRSV attachment (Fig. 5A and B), suggesting that FcRn is not an adsorptive receptor for PRRSV. A great deal of literatures have exhibited that HS and CD169 are adsorption receptors for PRRSV (16, 61). Our study suggested that FcRn is not a receptor for PRRSV internalization (Fig. 5C and D), but CD169 (15), HSPA8 (62), and MYH9 (63) are involved in viral internalization. Further investigation evidenced that nsp2 as the marker of RTCs was undetectable in FcRn-KO MARC-145 cells, which was consistent with observations in *mkCD163*-KO cells and other reports (24, 30). The phenomena suggest that the PRRSV did not successfully release genomic RNA into the cytoplasm in FcRn-KO cells and that FcRn might be involved in virus uncoating and genome release. The internalized PRRSV virions enter CD163-positive FcRn-positive early endosomes (Fig. 6A and B; Fig. S5A, and S5B) where the interaction between CD163 and PRRSV glycosylated membrane proteins (28, 30, 50) and the interaction between FcRn and M protein occur (Fig. 6C through E), providing a possible molecular basis for viral nucleocapsid exposure. CD163 expression confers HEK-293T and PK-15 (nonpermissive cells) susceptibility to PRRSV (27, 52, 64), and overexpression of FcRn alone in the two type cells does not result in successful PRRSV infection (Fig. S1B and C), indicating that CD163 interacting with viral envelope proteins to expose the nucleocapsid, which is a prerequisite for the subsequent interaction between FcRn and nucleocapsid and genome release. Due to limitations, earlier studies did not consider the role of FcRn when using these non-permissive cells to explore the receptor function of CD163 (27, 52, 64). Both PK-15 and HEK-293T cells express FcRn (35, 65), so we believe that the endogenous FcRn also plays a critical role when PRRSV infects HEK-293T and PK-15 cells expressing CD163. Despite the facts that the expression of CD163 from multiple species conferred non-permissive cells susceptible to PRRSV (14) and that complementing FCGRT from different species in *FCGRT*-KO MARAC-145 cells recovered viral proliferation (Fig. 4D and E), there are currently no reports of PRRSV cross-species transmission. It is worth mentioning that MARC-145 and Vero, both African green monkey kidney cells, express CD163 (14) and FcRn (Fig. S6), but no productive viral infection was observed in Vero cells (66). BHK-21 cells transfected with mkCD151 are susceptible to PRRSV (19), but COS-7 and Vero cells expressing endogenous CD151 did not support PRRSV replication (19, 21, 66). These puzzling questions deserve further exploration, and these studies also indicate that the successful infection of cells by PRRSV requires the synergy of multiple receptors and host factors. Whether there are other host factor (s) that interacts with FcRn and promotes PRRSV infection is something we must clarify in the future. Due to the lack of small animals for studying PRRSV pathogenicity and infection, Cui et al. developed genetically modified mice overexpressing pCD163, pCD169, and mkCD151 and found that the mice could not be infected by PRRSV, but the alveolar macrophages isolated from the mice could be successfully infected by PRRSV, which might be the result that the innate immune response of mice quickly cleared PRRSV (67), indicating that the environment of viral infection *in vivo* is more complicated.

**Fig 7:**
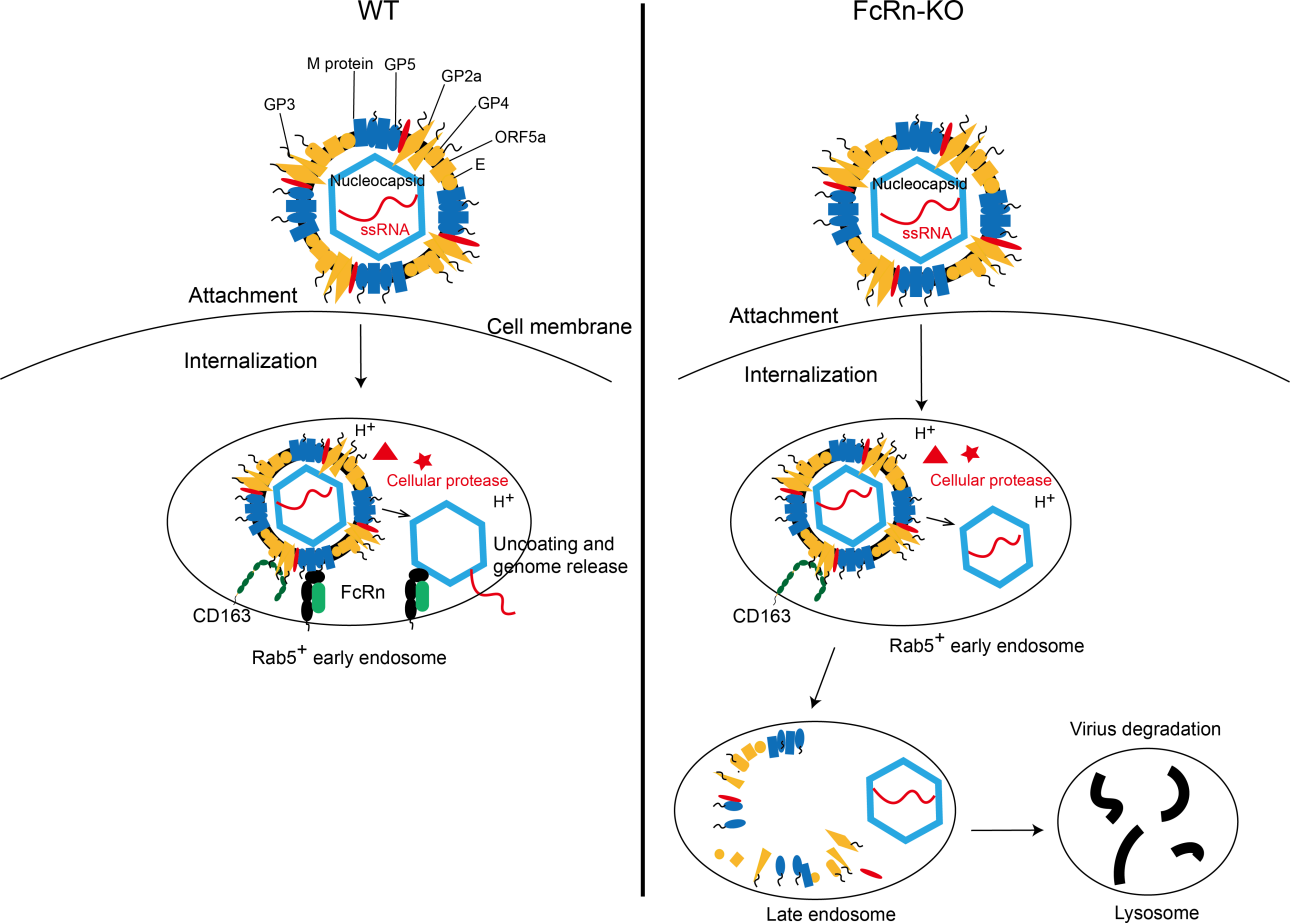
Schematic model of the role of FcRn in PRRSV uncoating and genome release. In WT MARC-145 cells or PAMs, after PRRSV virions internalize into early endosomes, the interaction between CD163 and GP2a, 3, 4, five and the interaction between FcRn and M protein have the nucleocapsid exposed. Subsequently, FcRn interacts with nucleocapsid to mediate uncoating and the release of genome RNA. Finally, the viral genome initiates its replication. In FcRn-KO MARC-145 cells or PAMs, after virions internalize into early endosomes, there is only the interaction between CD163 and viral GP2a, 3, 4, 5. Due to the lack of FcRn, virus uncoating and the release of genome RNA were blocked in the early endosomes. Subsequently, nucleocapsids were forced to the late endosomes and finally to the lysosomes, where they were degraded.

Studies have shown that human FcRn directly interacts with echovirus 6 or 18 (non-enveloped virus) nucleocapsid proteins (VP1, VP2, and VP3) in early endosomes, leading to virions conformational changes and subsequent uncoating (32, 35). Considering that FcRn interacts with PRRSV nucleocapsid protein in early endosomes (Fig. 6A through F), we believe that FcRn is very likely to be the PRRSV uncoating receptor, but this view requires more trustworthy proof to prove. Weakly acidic environment (66, 68), cathepsin E (69, 70), unnamed serine protease (70), and calpain 1 (30) were also thought to play a role in PRRSV uncoating. According to the previous method (35), we tried to express FcRn and CD163 extracellular domains through HEK-293T cells, but the expression levels were too low to obtain purified soluble proteins. PRRSV uncoating might be more intuitively visualized by incubation of receptor-modified liposomes with the virus and the use of transmission electron microscopy. Cryo-electron microscopy should be used to analyze the atomic structure of the receptor-PRRSV complex and identify the interaction domains and key amino acid sites, which will lay the foundation for FcRn as an anti-PRRSV target in the future. Pigs with CD163 SRCR5 deletion not only exhibited resistance to PRRSV infection but also maintained the normal physiological function of CD163 (24). A Chinese research team has developed *FCGRT*-KO pigs (71). Future studies should use these gene-edited pigs in challenge experiments to investigate the role of FcRn in PRRSV infection at the animal level. Based on the results stated above, we propose a model to depict the role of FcRn in PRRSV uncoating and genome release (Fig. 7). The movement paths of uncoated PRRSV virions in *CD163 SRCR5*-KO MARC-145 cells have been described (30), and the trajectories of PRRSV virions in FcRn-KO MARC-145 cells should be elucidated in future study.

In conclusion, FcRn was identified as a PRRSV uncoating factor for the first time. Our findings are beneficial for the understanding of PRRSV infection and provide insights into the development of novel effective vaccines and antiviral drugs for PRRSV.

## MATERIALS AND METHODS

### Cells and viruses

MARC-145, HEK-293T, PK-15, and Vero cells were cultured in Dulbecco’s modified Eagle’s medium (DMEM; Hyclone, Logan, USA) containing 10% fetal bovine serum (FBS; Gibco, CA, USA) and 1% penicillin-streptomycin. Porcine alveolar macrophages (PAMs) were obtained from lung lavage samples from the lungs of 4-week-old PRRSV-negative piglets and cultured in RPMI 1640 medium supplemented with 10% FBS and 1% penicillin-streptomycin. All cells were cultured at 37℃ with 5% CO_2_. MARC-145, HEK-293T, PK-15, and Vero cells were stored in our laboratory (39, 65, 72). PRRSV-2 strain XJ (GenBank accession no. OR102496.1) and FJ (GenBank accession no. OR102497.1) are highly pathogenic PRRSV (Sublineage 8.7), isolated from the blood of pigs suffering from high fever syndrome in China in 2018 and 2019 in our laboratory, respectively. PRRSV-2 strain SH (GenBank accession no. OR102498.1) is NADC30-like PRRSV (Sublineage 1.8), which was isolated from the lung of a pig suffering from difficulty breathing in China in our laboratory in 2020. PRRSV-1 strain SH2022 was isolated by our laboratory from pig serum from Shanghai in 2022. PRRSV-2 vaccine strains JXA1-R (sublineage 8.7) and R98 (sublineage 5.1) were purchased from Wuhan Keqian Biological Co., Ltd. (Wuhan, China) and China Animal Husbandry Industry Co., Ltd. (Beijing, China), respectively.

### Antibodies and reagents

Affinipure rabbit polyclonal antibody (pAb) against porcine FCGRT cytoplasmic tail (pFCGRT-CT) was kept in our laboratory (39). Rabbit anti-FcRn α chain pAb (catalog no. 16190-1-AP), mouse anti-β2m monoclonal antibody (mAb; catalog no. 66207-1-Ig), mouse IgG2b isotype control (catalog no. 66360-3-Ig), mouse anti-EEA1 mAb (catalog no. 68065-1-Ig), and CoraLite647-conjugated affinipure F(ab′)2 fragment goat anti-mouse IgG (H + L) (catalog no. SA00014-10) were purchased from proteintech (Wuhan, China). Rabbit pAb against Cas9 (catalog no. A14997), mouse or rabbit mAb against FLAG (catalog no. AE092), HA (catalog no. AE008), Myc (catalog no. AE070), mouse anti-glyceraldehyde phosphate dehydrogenase (GAPDH) mAb (catalog no. AC002), horseradish peroxidase (HRP)-labeled goat anti-mouse (catalog no. AS003) or rabbit (catalog no. AS014) IgG (H + L), ABflo 594-conjugated goat anti-rabbit (catalog no. AS039) or mouse (catalog no. AS054) IgG (H + L), and rabbit IgG isotype control (catalog no. AC042) were purchased from ABclonal (Wuhan, China). Mouse mAb against CD163 (catalog no. GTX42364), rabbit anti-PRRSV N protein pAb (catalog no. GTX135351), rabbit anti-PRRSV M protein pAb (catalog no. GTX129063), mouse anti-PRRSV M protein mAb (catalog no. GTX634651), rabbit anti-PRRSV GP5 pAb (catalog no. GTX129062), rabbit anti-PRRSV GP3 pAb (catalog no. GTX129272), rabbit anti-PRRSV nsp1α pAb (catalog no. GTX133695), and fluorescein isothiocyanate (FITC)-conjugated rabbit anti-pig IgG (catalog no. GTX26773) were purchased from GeneTex (San Antonio, USA). Pig IgG (catalog no. bs-0309P) was purchased from Bioss (Beijing, China). Mouse mAb against PRRSV nsp2 are generous gifts from Prof. Changjiang Weng of Harbin Veterinary Research Institute, Chinese Academy of Agricultural Sciences, China (73). Mouse mAb against PRRSV N protein and porcine sera against PRRSV strain PC were liberal gifts from Dr. Jian Li of Huazhong Agricultural University, China. The ORF1a and ORF1b of the classic PRRSV strain SP (GenBank accession no. AF184212.1) and the ORF2-ORF7 of the HP-PRRSV strain GD (GenBank accession no. EU825724.1) were recombined into a chimeric vaccine strain PC. The porcine sera against PC were purified by protein G following the manufacturer’s instructions (catalog no. SA020GC05; Smart-Lifesciences, Changzhou, China) and could cross-react with PRRSV-2 strain FJ.

Cell lysis buffer for Western blot and Co-immunoprecipitation was purchased from Beyotime (RIPA; Shanghai, China). Lipofectamine 3000 transfection reagent (catalog no. L3000008) was purchased from Invitrogen (Carlsbad, USA). Cell Counting Kit-8 (CCK-8; catalog no. BS350A) was purchased from Biosharp (Hefei, China). 4′,6-Diamidino-2-phenylindole dihydrochloride (DAPI; catalog no. C0065) was purchased from Solarbio (Beijing, China).

### Construction of plasmids

Lentiviral expression plasmids pLVX-porcine FCGRT-shRNA-ZsGreen were generated by cloning the annealed shRNAs sequences into empty vector pLVX-shRNA-ZsGreen (Addgene, Cambridge, USA). The shRNAs against pFCGRT listed in Table 1 were designed on the website (https://rnaidesigner.thermofisher.com/rnaiexpress/sort.do). Lentiviral expression plasmids pLV-mkFCGRT-sgRNA-EGFP, pLV-mkB2M-sgRNA-EGFP, and pLV-mkCD163-sgRNA-EGFP were constructed by cloning the annealed sgRNAs sequences into BbsI (NEB, Ipswich, USA) site in empty vector pLV-sgRNA-EGFP (Addgene), respectively. The sgRNAs targeting the corresponding gene were designed on the website (http://crispor.tefor.net/crispor.py) and the PCR primers used to amplify the sgRNA-recognizing regions are listed in Table 2. The pFCGRT, mkFCGRT, and mkB2M coding sequence (CDS) were amplified by PCR of cDNA from PAMs or MARC-145 cells using the primers listed in Table 3 and then cloned into empty vector pCMV-Tag2B (Addgene) to produce pCMV-Tag2B-pFCGRT, pCMV-Tag2B-mkFCGRT, and pCMV-Tag2B-mkB2M, respectively. The human (h) FCGRT (GenBank accession no. U12255.1), mouse (m) FCGRT (GenBank accession no. BC003786.1), rat (r) FCGRT (GenBank accession no. BC061975.1), bovine (b) FCGRT (GenBank accession no. BC102159.1), and dog (d)

**TABLE 1:** shRNAs used in this study

Primer name*^a^*	Sequence (5′−3′)
shRNA-1-F	GATCGGAACAAGCAGAAGCTCTTTCTTCAAGAGAGAAAGAGCTTCTGCTTGTTCCTTTTTG
shRNA-1-R	AATTCAAAAAGGAACAAGCAGAAGCTCTTTCTCTCTTGAAGAAAGAGCTTCTGCTTGTTCC
shRNA-2-F	GATCGCGAGGAGTTTATGAAGTTCGTTCAAGAGACGAACTTCATAAACTCCTCGCTTTTTG
shRNA-2-R	AATTCAAAAAGCGAGGAGTTTATGAAGTTCGTCTCTTGAACGAACTTCATAAACTCCTCGC
shRNA-3-F	GATCGGTCGTCGCTAACAGTCAAGATTCAAGAGATCTTGACTGTTAGCGACGACCTTTTTG
shRNA-3-R	AATTCAAAAAGGTCGTCGCTAACAGTCAAGATCTCTTGAATCTTGACTGTTAGCGACGACC
shRNA-4-F	GATCGCTTCCTACTGCTCTTGATCGTTCAAGAGACGATCAAGAGCAGTAGGAAGCTTTTTG
shRNA-4-R	AATTCAAAAAGCTTCCTACTGCTCTTGATCGTCTCTTGAACGATCAAGAGCAGTAGGAAGC
Non-target-F	GATCCAACAAGATGAAGAGCACCAATTCAAGAGATTGGTGCTCTTCATCTTGTTGTTTTTG
Non-target-R	AATTCAAAAACAACAAGATGAAGAGCACCAATCTCTTGAATTGGTGCTCTTCATCTTGTTG

^*a*^ F, forward primer; R, reverse primer.

**TABLE 2:** sgRNAs used in this study

Primer group and name*^a^*	Sequence (5′−3′)
Primers for sgRNAs	
mkFCGRT-sgRNA-F	CACCGCCTGAGCTACGATAGCCTG
mkFCGRT-sgRNA-R	AAACCAGGCTATCGTAGCTCAGGC
mkB2M-sgRNA-F	CACCGACCCAGACACATAGCAATTC
mkB2M-sgRNA-R	AAACGAATTGCTATGTGTCTGGGTC
mkCD163-sgRNA-F	CACCGAACGGTGTGTAATAATGGC
mkCD163-sgRNA-R	AAACGCCATTATTACACACCGTTC
Primers for PCR amplifIcation of CRISPR/Cas9 recognizing regions	
mkFCGRT-genome-F	GCCACTGCAATCTAGTTCCCCGC
mkFCGRT-genome-R	CCTTCCACTCCAGGTTTCCACGG
mkB2M-genome-F	AAGTGAAATACCCTGGCAATATTAAT
mkB2M-genome-R	CTGAATGCTCCACTTTCCCCATT
mkCD163-genome-F	GTCTTAATCCATTGTTGCAGGAGGAAC
mkCD163-genome-R	AAATGCGTCCAGAACCTGCACTG

^*a*^ F, forward primer; R, reverse primer.

**TABLE 3:** Primers for plasmid constructs and qPCR

Primer group and name*^a^*	Sequence (5′−3′)
Primers for protein expression	
pFCGRT-F	ATGCGGGTCCCCCGGCCTCA
pFCGRT-R	AGATTCAGCATCCTTGGCCAGGTCGG
mkFCGRT-F	ATGAGGGTCCCGCGGCCT
mkFCGRT-R	GGCAGTGGCTGGGATCACATTTAT
mkB2M-F	ATGTCTCGCTCAGTGGCCTTAG
mkB2M-R	CAAAAGAATGTAAGACTTACCCCACT
mkCD163-F	ATGAGCAAACTCAGAATGGTGCTACTTGAAGACTCTGGAT
mkCD163-R	GTGTGCCTCAGAATGGCCTCCTGAGGAATTCATTAGGTCC
Primers for qPCR	
PRRSV-2 ORF7-F	CCAGCCAGTCAATCAGCTGT
PRRSV-2 ORF7-R	CACTAGGGGTAAAGTGATGCCT
mkGAPDH-F	TCATGACCACAGTCCACGCC
mkGAPDH-R	GGATGACCTTGCCCACAGCC
PRRSV-1 ORF7-F	TGGGGAATGGCCAGCCAGT
PRRSV-1 ORF7-R	CTGGATGAAAGCGACGCAGT
pGAPDH-F	AGTGGACATTGTCGCCATCAAT
pGAPDH-R	GAAGACACCAGTGGACTCCACA

^*a*^ F, forward primer; R, reverse primer.

FCGRT (GenBank accession no. XM_005616309.4) CDS were synthesized (Tsingke, Wuhan, China) and cloned into pLV-CMV-FLAG-HygR (Addgene) to generate FLAG-tagged expression plasmids. The pFCGRT, mkFCGRT, and mkB2M CDS were cloned into the pLV-CMV-FLAG-HygR, and the three CDS PAM sequences recognized by CRISPR/Cas9 were synonymously mutated. The mkCD163 CDS (GenBank accession no. JF753553.1) was synthesized and cloned into pLV-CMV-HA-HygR to yield an HA-tagged expression plasmid.

### Lentivirus generation

6-well plates HEK-293T cells were cotransfected with pMD2.G (Addgene), a plasmid expressing vesicular stomatitis virus glycoprotein (VSV-G), psPAX2 (Addgene), a lentiviral packaging plasmid, and recombinant lentiviral expression plasmids mentioned in “Construction of plasmids,” above, at a ratio of 1:3:4 using Lipofectamine 3000 according to the manufacturer’s instructions. The supernatants were harvested at 48 h after cotransfection. 2 mL of complete nutrient solution then was added to the original wells. The supernatants were collected after 24 h and then mixed with the first harvested supernatants, followed by filtering with a 0.45-µm filter (Millipore, Darmstadt, Germany). The lentiviral filtrates were immediately used for subsequent experiments.

### shRNA-mediated FcRn knockdown

PAMs were infected with recombinant lentivirus expressing shRNAs against FCGRT or control lentivirus expressing non-target shRNA. After 36 h, PAMs were collected for Western blot analysis to detect the knockdown efficiency of FCGRT. The FCGRT-knockdown PAMs or control cells were applied for subsequent PRRSV infection experiments. The infected cells were harvested for detection of PRRSV proteins expression by Western blot using indicated antibodies or PRRSV titers by TCID_50_ assay, respectively.

### FcRn overexpression

MRAC-145 cells were transfected with 1.5 µg pCMV-Tag2B-FCGRT or empty vector using Lipofectamine 3000 according to the manufacturer’s instructions. After 36 h, the cells were applied for subsequent PRRSV infection experiments. The infected cells were collected for detection of PRRSV N protein expression by Western blot using indicated antibodies or PRRSV titers by TCID_50_ assay, respectively.

### Antibodies and IgG blocking assay

The effects of FcRn antibodies on PRRSV infection were detected according to a previous study (74). Briefly, PAMs or MARC-145 cells on 24-well plates were incubated with 200 µL medium containing indicated concentrations of antibodies or its isotype IgG at 37°C for 1 h before infection. After washes, PRRSV strain FJ (MOI of 1.0) was incubated with the cells at 37℃ for 1 h. After another round of washes, the cells were again incubated with a medium containing the corresponding antibodies or its isotype IgG at 37°C. At 30 h after infection, the infected cells were harvested to determine PRRSV N protein expression using Western blot with anti-PRRSV N pAb or virus titers using TCID_50_ assay. The effects of IgG from different species on PRRSV infection were carried out according to the same method as above.

### Cytotoxicity assay

Cell viability was determined by CCK-8 assay. In brief, MARC-145 cells or PAMs were seeded onto 96-well plates with opaque walls. FcRn antibodies or IgG were added at indicated concentrations. At 24 h post-addition, 100 µL of CCK-8 solution was added directly into each well and incubated with the cells for 1 h at 37°C. The optical density at 450 nm (OD_450_) was measured using a microplate reader (Thermo Fisher Scientific, Waltham, USA). The cell viability of the experimental group was calculated according to the manufacturer’s instructions.

The cell viability of PAMs treated with shRNA and three single-gene KO MARC-145 cells was calculated according to the same method as above.

### Knockout cell lines establishment and validation

A single clone of MARC-145 cells stably expressing Cas9 (MARC-Cas9) was constructed by infection with lentiviruses packaged with lentiviral plasmid pLV-Cas9-puroR (Addgene) and selection by 10 μg/mL puromycin (catalog no. ST551; Beyotime). Subsequently, *FCGRT*-KO, *B2M*-KO, and *CD163*-KO MARC-145 cell lines were established by infection with lentiviruses packaged with lentiviral plasmids expressing sgRNAs targeting the corresponding genes. The sgRNAs sequence is listed in Table 2. Single cells were seeded in 96-well plates with the limiting dilution method. After selection with EGFP and expansion, the cell clones in each well were transferred to 12-well plates for further culture. The clones were screened for protein expression by Western blot analysis using indicated antibodies, and the clones lacking corresponding gene expression were expanded and preserved. After extracting cellular genomic DNA, the three DNA fragments of target loci were amplified by PCR with three primer pairs listed in Table 2. The purified PCR products were sequenced (Tsingke, Wuhan, China), and the insertions and deletions (indels) within three genes caused by CRISPR/Cas9 were analyzed.

### Construction of complemented cell lines

The lentiviruses packaged with recombinant lentiviral plasmids mentioned in “Construction of plasmids,” above, were introduced into corresponding KO cell lines. *FCGRT*-KO + mkFCGRT, *FCGRT*-KO + pFCGRT, *FCGRT*-KO + hFCGRT, *FCGRT*-KO + mFCGRT, *FCGRT*-KO + rFCGRT, *FCGRT*-KO + bFCGRT, *FCGRT*-KO + dFCGRT, *FCGRT*-KO + mkFCGRT-tailless, *FCGRT*-KO + pFCGRT-tailless, and *B2M*-KO + mkB2 M cells were selected with 1,000 µg/mL Hygromycin B (catalog no. ST1389; Beyotime) for 72 h, and *FCGRT*-KO + empty vector cells were used as control. The expression of complemented genes was detected by Western blot using indicated antibodies.

### Brightfield imaging of CPE and viral TCID_50_ assay

For bright-field images, *FCGRT*-KO, *B2M*-KO, *CD163*-KO, and WT MARC-145 cells were infected with PRRSV strain FJ (MOI of 1.0). Cells were imaged at 48 hpi, and the cytopathic effect (CPE) was read by the inverted microscope (Olympus, Tokyo, Japan). For the TCID_50_ assay, MARC-145 cells or PAMs were seeded in 96-well plates and then inoculated with 100 µL serial 10-fold dilutions of PRRSV samples in eight replicates. The plates were incubated for 3–5 days before virus titers were calculated. PRRSV titers were expressed as 50% tissue culture infective dose per milliliter (TCID_50_/mL) using the Reed-Muench method.

### RNA extraction and quantitative PCR (qPCR)

Total RNA was extracted from cultured cells with Trizol reagent (Invitrogen, Carlsbad, USA) according to standard protocols and subsequently reverse transcribed into cDNA using a PrimeScript RT reagent kit (TaKaRa, Dlian, China) in accordance with the manufacturer’s instructions. SYBR green (TaKaRa) real-time PCR was performed using the CFX96 system (BIO-RAD, Hercules, USA). Relative mRNA expression levels were normalized to the expression of GAPDH. Absolute quantitative mRNA levels were calculated using standard curves. The primers used for qPCR are listed in Table 3.

### Virus attachment and internalization assays

Attachment assay: *FCGRT*-KO, *B2M*-KO, and WT MARC-145 cells cultured in 12-well plates were pre-chilled at 4°C for 1 h. After washing twice with cold PBS, PRRSV strain FJ (MOI = 1) was incubated with the cells at 4°C for 2 h (to permit attachment but prevent internalization). After washing twice with cold PBS to remove the free virus, the cells were collected, and RNA was extracted for qPCR analysis. Internalization assay: after incubation at 4°C for 2 h, the 12-well plates were directly placed at 37°C for 2 h to allow virus entry. After twice washes, the cells were collected for RNA extraction and qPCR analysis.

### Western blot analysis

The prepared samples were electrophoresed using 10% or 12% SDS-PAGE (EpiZyme, Shanghai, China). Proteins were transferred onto polyvinylidene difluoride membranes (PVDF; Millipore, Darmstadt, Germany). The membranes were blocked with 3% skim milk at room temperature (RT) for 2 h and then stained at 4°C overnight using the indicated primary antibodies (1:1,000 dilution). After three washes with TBST (pH 7.4 TBS with 0.1% Tween 20), the membranes were incubated with appropriate HRP-labeled secondary antibodies (1:5,000 dilution) at RT for 2 h. After another round of washes, proteins bands were visualized and analyzed using enhanced chemiluminescence (ECL) kit (catalog no. RM00021; ABclonal, Wuhan, China) and ChemiDoc XRS + system (BIO-RAD, Hercules, USA).

### Co-immunoprecipitation

Cells were collected and lysed with RIPA (pH 6.0 or 7.4) for 20 min on ice. The lysates were incubated with appropriate antibodies at 4°C for 2 h, followed by precipitation with protein A/G magnetic beads (catalog no. B23201; Selleck, Houston, USA) for 4 h at 4℃, The samples were washed with PBST (PBS with 0.5% Tween 20; pH 6.0 or 7.4) for six times and detected by Western blot using the indicated antibody.

### Immunofluorescence assay and confocal microscopy

MARC-145 cells or PAMs were seeded in 24-well plates or confocal dishes and then inoculated with PRRSV strain FJ (MOI = 1). After the indicated time of infection, the cells were fixed with 4% paraformaldehyde for 30 min at RT and permeabilized with 0.1% Triton X-100 at RT for 20 min. After three times washes with PBS, the cells were incubated with indicated primary antibodies (1:100 dilution) for 2 h at 37°C. Cells then were washed with PBS, followed by incubation with appropriate fluorescently labeled secondary antibodies (1:200 dilution) for 1 h at 37°C. Cell nuclei were stained with DAPI for an additional 15 min. The fluorescent images were acquired by a confocal laser scanning microscope (LSM800; Carl Zeiss AG, Oberkochen, Germany). Manders’ overlap coefficient (>0.6) and Pearson’s correlation coefficient (>0.5) are considered to represent the true degree of colocalization and interaction, respectively (75).

### Statistical analyses

GraphPad Prism 5 software (San Diego, USA) was used for data analysis using a two-tailed unpaired t-test or one-way analysis of variance (ANOVA). Statistical significance in the figures is indicated by asterisks (*P < 0.05; **P < 0.01; ***P < 0.001; ****P < 0.0001; ns, not significant [*P* > 0.05]).

## Data Availability

The data underlying this article are available in this article and its supplemental material.

## SUPPLEMENTAL MATERIAL

Supplemental material jvi.01218-24-s0001.doc Figures S1 to S6.

ASM does not own the copyrights to Supplemental Material that may be linked to, or accessed through, an article. The authors have granted ASM a non-exclusive, world-wide license to publish the Supplemental Material files. Please contact the corresponding author directly for reuse.
