# Supplementary material for: The neonatal Fc receptor (FcRn) is required for porcine reproductive and respiratory syndrome virus uncoating
Source: J Virol. 2024 Dec 9;99(1):e01218-24. doi: 10.1128/jvi.01218-24 (PMC11784455; doi:10.1128/jvi.01218-24)
Supplement: Supplemental material — Figures S1 to S6. [file jvi.01218-24-s0001.doc]

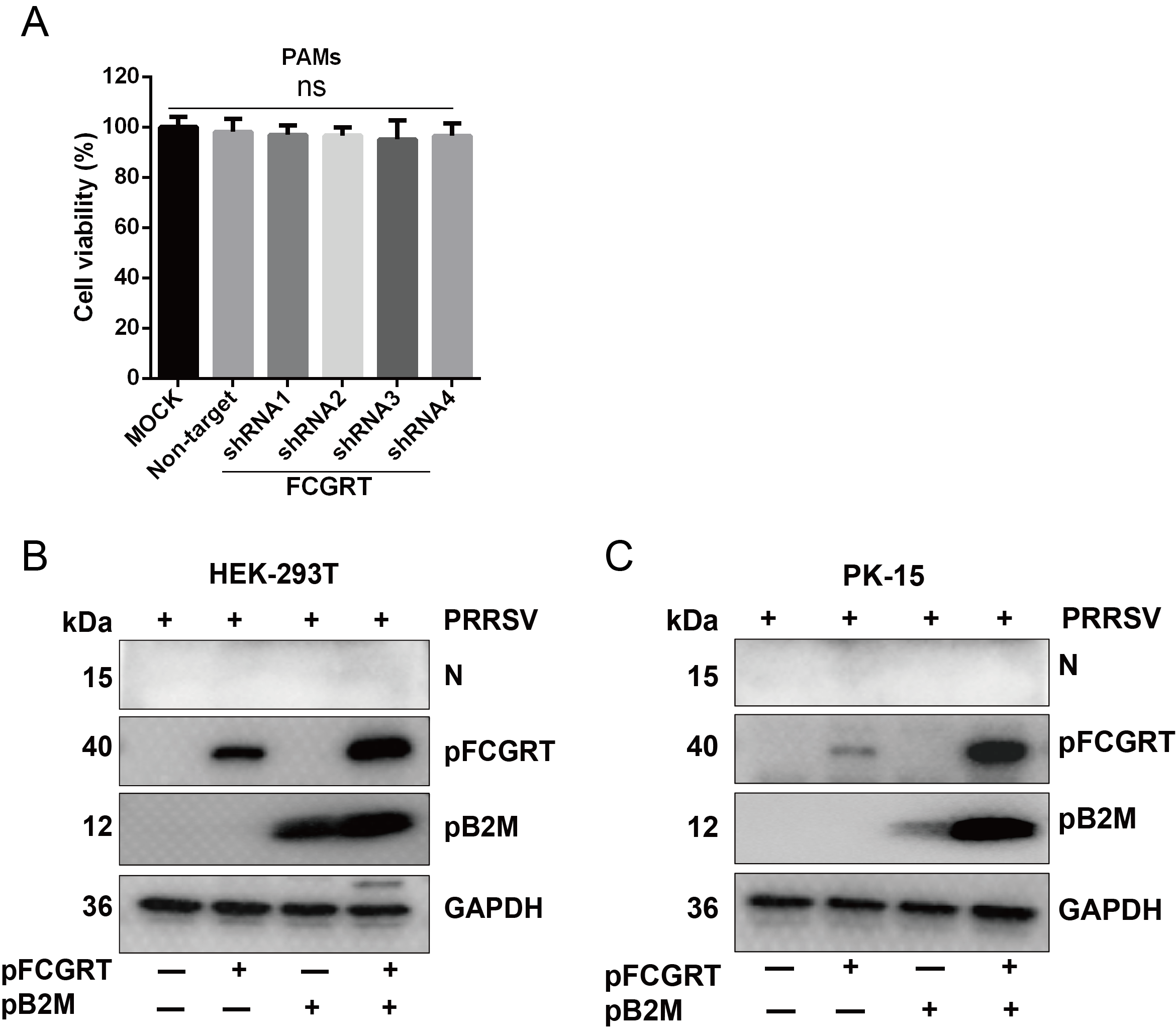


**Figure S1.** (A) Determination of the cytotoxicity of shRNAs to PAMs. Cells were transduced with recombinant lentivirus expressing *FCGRT*-specific shRNAs or control lentivirus expressing non-target shRNA. At 72 h after transduction, cells viability was measured by CCK-8 assay. ns, not significant (P > 0.05). (B and C) Overexpression of FcRn in HEK-293T and PK-15 cells does not result in successful PRRSV infection. HEK-293T (B) or PK-15 (C) cells were transfected with the eukaryotic expression plasmids encoding FLAG-tagged pFCGRT and Myc-tagged pB2M in the way as shown in this picture for 24 h, followed by PRRSV strain FJ (MOI of 1.0) infection. At 48 hpi, cells were collected to determine the expression of viral N protein and FcRn by Western blot using anti-N protein, anti-FLAG, anti-Myc, and anti-GAPDH antibodies. Data represent means ± SD from three independent experiments. Significant differences from results with the control group are indicated as follows: ns, not significant (P > 0.05).


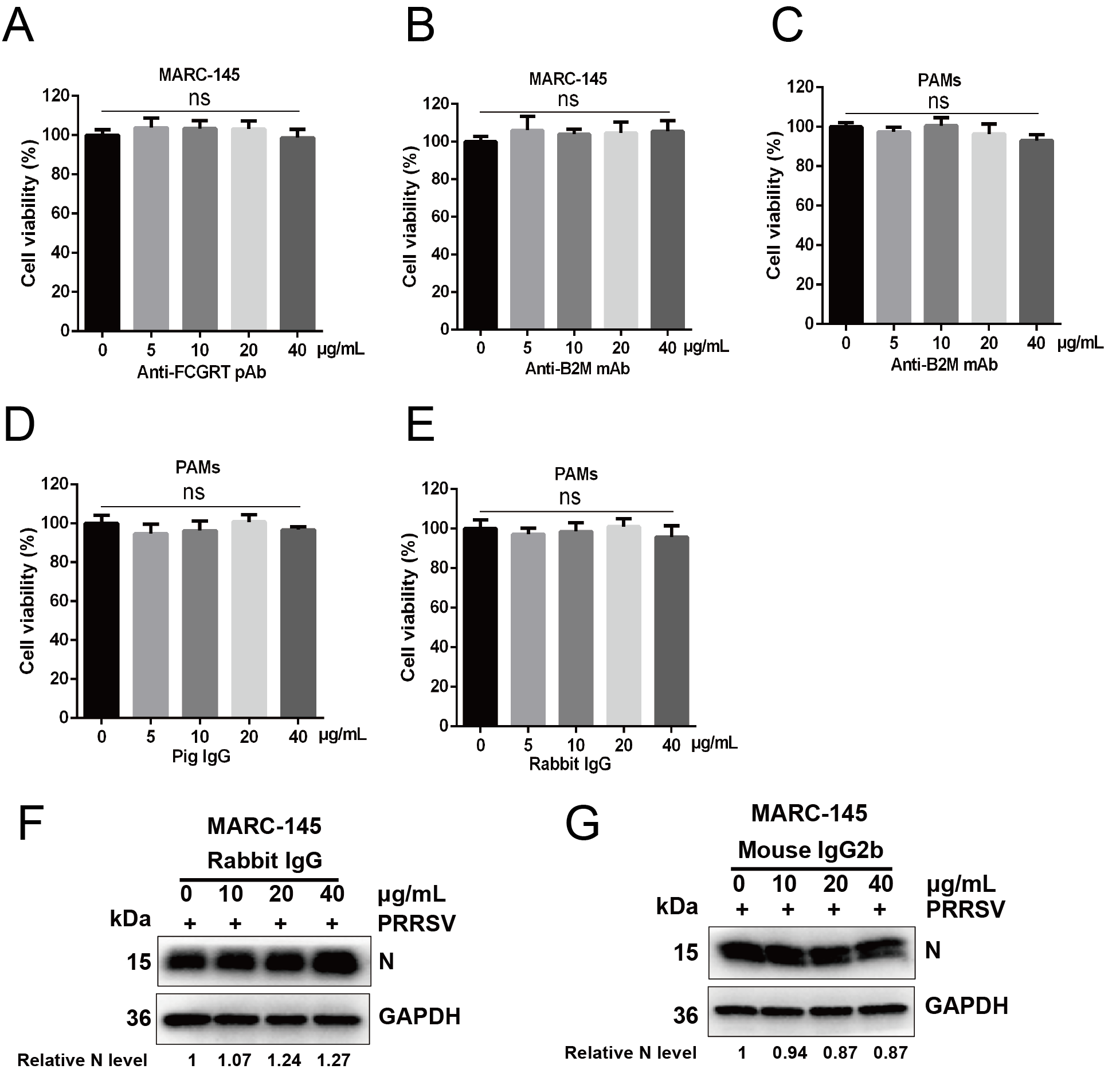


**Figure S2.** Determination of the cytotoxicity of specific anti-FcRn IgG or normal IgG to indicated cells and the effects of IgG on PRRSV proliferation in MARC-145 cells. (A to E) The indicated cells (MARC-145 cells, PAMs) were dealt with indicated concentrations (5, 10, 20, 40 *μ*g/mL) FcRn antibodies (A to C) or IgG (D and E) from different species (pig, rabbit) for 30 h. Cells viability was measured by CCK-8 assay. ns, not significant (P > 0.05). (F and G) PRRSV proliferation in MARC-145 cells couldn't be inhibited by rabbit or mouse IgG. MARC-145 cells were pretreated with rabbit IgG or mouse IgG2b at the indicated concentrations (10, 20, 40 *μ*g/mL) for 1 h at 37℃ prior to PRRSV strain FJ (MOI 1) infection. The infected cells were cultured in the presence of IgG and then harvested at 30 hpi to determine viral N protein expression by Western blot with anti-PRRSV N pAb. ImageJ software was used to analyze the relative levels of PRRSV N protein in comparison with control group, and the ratios are displayed as fold changes below the images. Data represent means ± SD from three independent experiments. Significant differences from results with the control group are indicated as follows: ns, not significant (P > 0.05).

**
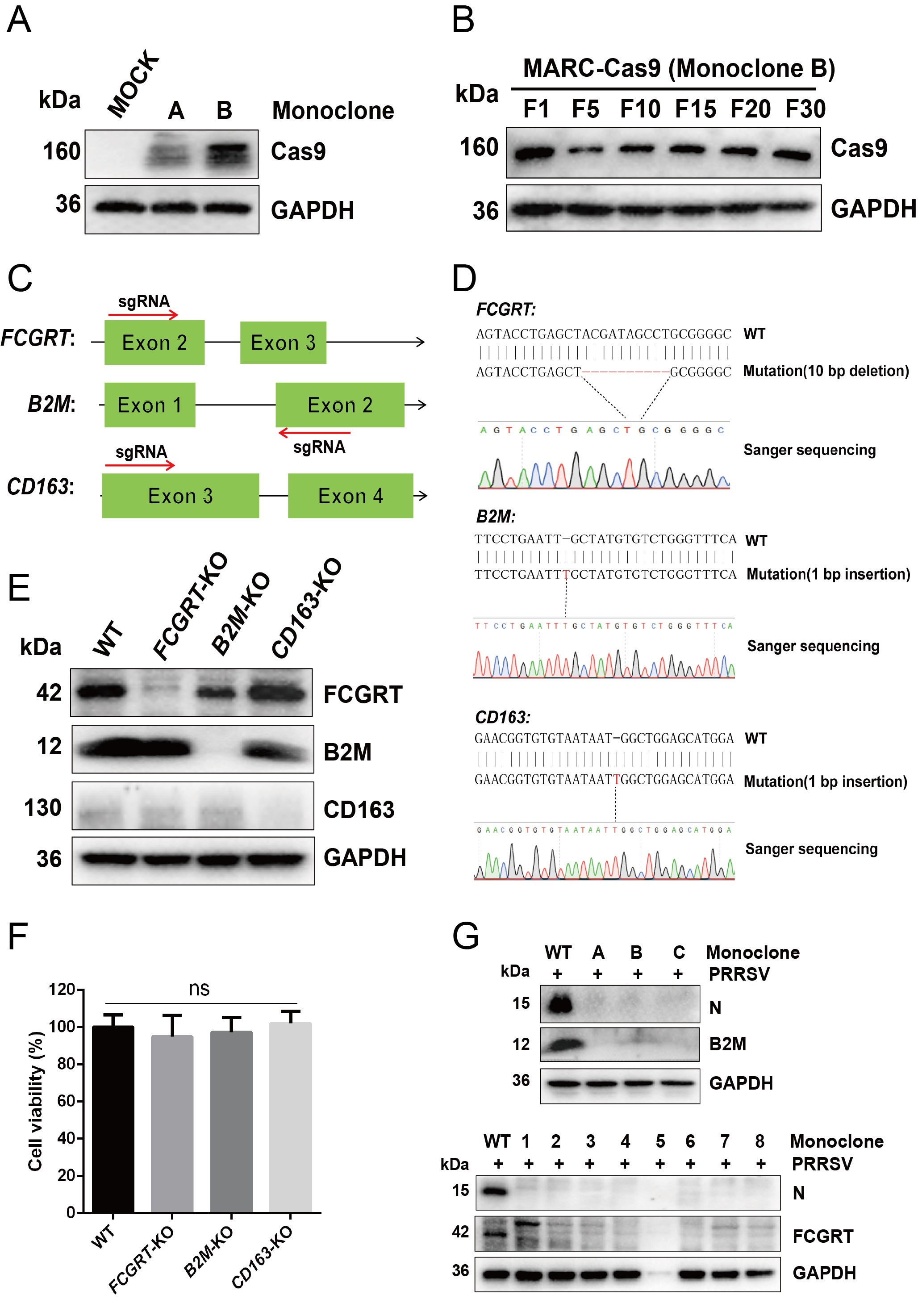
**

**
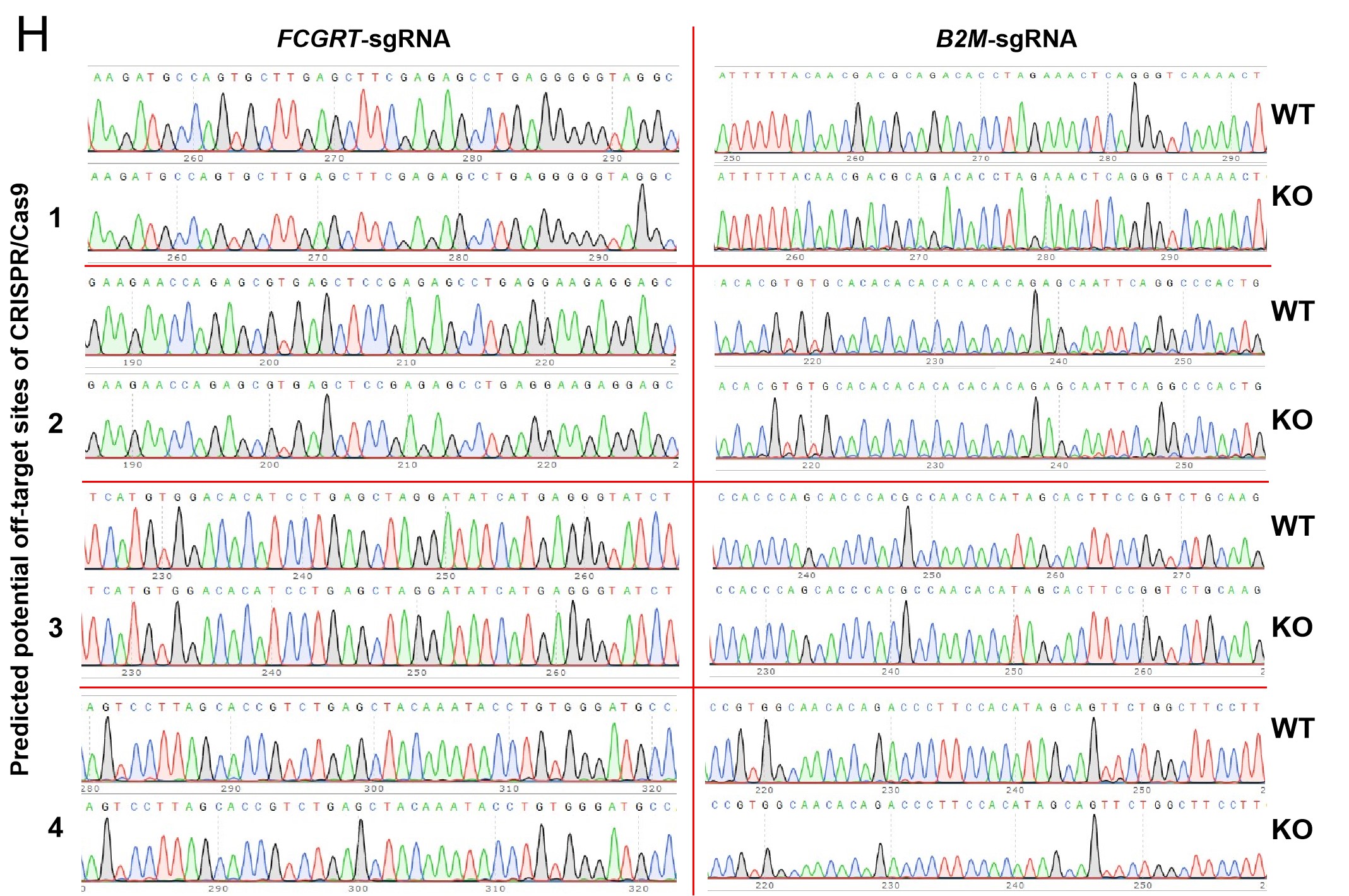
**

**Figure S3.** (A to E) Construction of *FCGRT*-KO, *B2M*-KO, and *CD163*-KO MARC-145 cell lines. (A) MARC-145 cells were transduced with recombinant lentivirus expressing Cas9,,and then 48 h later cells were treated with 10 *μ*g/mL puromycin for another 48 h. After screening monoclonal cells using limiting dilution method, the Cas9 expression of clones were analyzed by Western blot with anti-Cas9 pAb. (B) The monoclonal cell B expressing Cas9 was serially passaged, and the cells were harvested at passages 1, 5, 10, 15, 20, and 30. The expression of Cas9 was detected by Western blot using anti-Cas9 pAb. (C) Schematic of sgRNAs targeting genes in MARC-145 cells. Green boxes represent exons of indicated genes, and red arrows represent the direction of the sgRNA recognition. (D) MARC-Cas9 cells (monoclone B; F1) were transduced with recombinant lentivirus expressing indicated sgRNAs. After monoclonal cells were selected with EGFP and expanded, the cells genomes were extracted, and sgRNAs targeting regions were amplified by PCR. The purified PCR products were sequenced, and the insertions and deletions (indels) compared to WT were analyzed. (E) The monoclonal cells containing frameshift mutations were cultured in 6-well plates until they reached full monolayer and then harvested to detect the expression of corresponding proteins by Western blot with anti-FCGRT pAb, anti-B2M mAb, and anti-CD163 mAb. (F) Cells viability of *FCGRT*-KO, *B2M*-KO, *CD163*-KO, and WT MARC-145 cells was determined by CCK-8 assay. ns, not significant (P > 0.05). (G) The *B2M*-KO and *FCGRT*-KO cells from different clones were infected with PRRSV strain FJ (MOI=1) for 30 h. The infected cells were collected to detect the B2M, FCGRT, and viral N protein expression by Western blot using anti-B2M mAb, anti-FCGRT pAb, and anti-N protein pAb. Data represent means ± SD from three independent experiments. Significant differences from results with the control group are indicated as follows: ns, not significant (P > 0.05). (H) *FCGRT*-KO, *B2M*-KO, and WT MARC-145 cells were subjected to genome extraction, predicted off-target sites were amplified by PCR using primers listed in Table S1, and the sanger sequencing results were shown.

**Table S1 PCR primers used to amplify potential off-target sites**

| primers | | Sequences (5’-3’) |
| --- | --- | --- |
| *FCGRT-*sgRNA | off-target-1-F | tggtgtcctccccaactcct |
|  | off-target-1-R | ccactcccacacaaagaacacc |
|  | off-target-2-F | gaccgtttggaaggctctgct |
|  | off-target-2-R | ggaagcaatcatagtggaagca |
|  | off-target-3-F | ttgggtgggaacacaggggc |
|  | off-target-3-R | tggaattgggcatagggaaggat |
|  | off-target-4-F | atagttctggaggctggatgtcc |
|  | off-target-4-R | caggaagtggcagaatgtagcc |
| *B2M*-sgRNA | off-target-1-F | tacaccccaccaacagtgtgt |
|  | off-target-1-R | agaagggcaaagggaactgca |
|  | off-target-2-F | cataacctctttgcaagacaggcc |
|  | off-target-2-R | cctgtccgacatttcagaccc |
|  | off-target-3-F | gtgagtggagcacaagtgggt |
|  | off-target-3-R | gtagaaagaggccgggagtcag |
|  | off-target-4-F | ctagggtttggtccctctgcc |
|  | off-target-4-R | aagaccctgtaccacaaggcc |


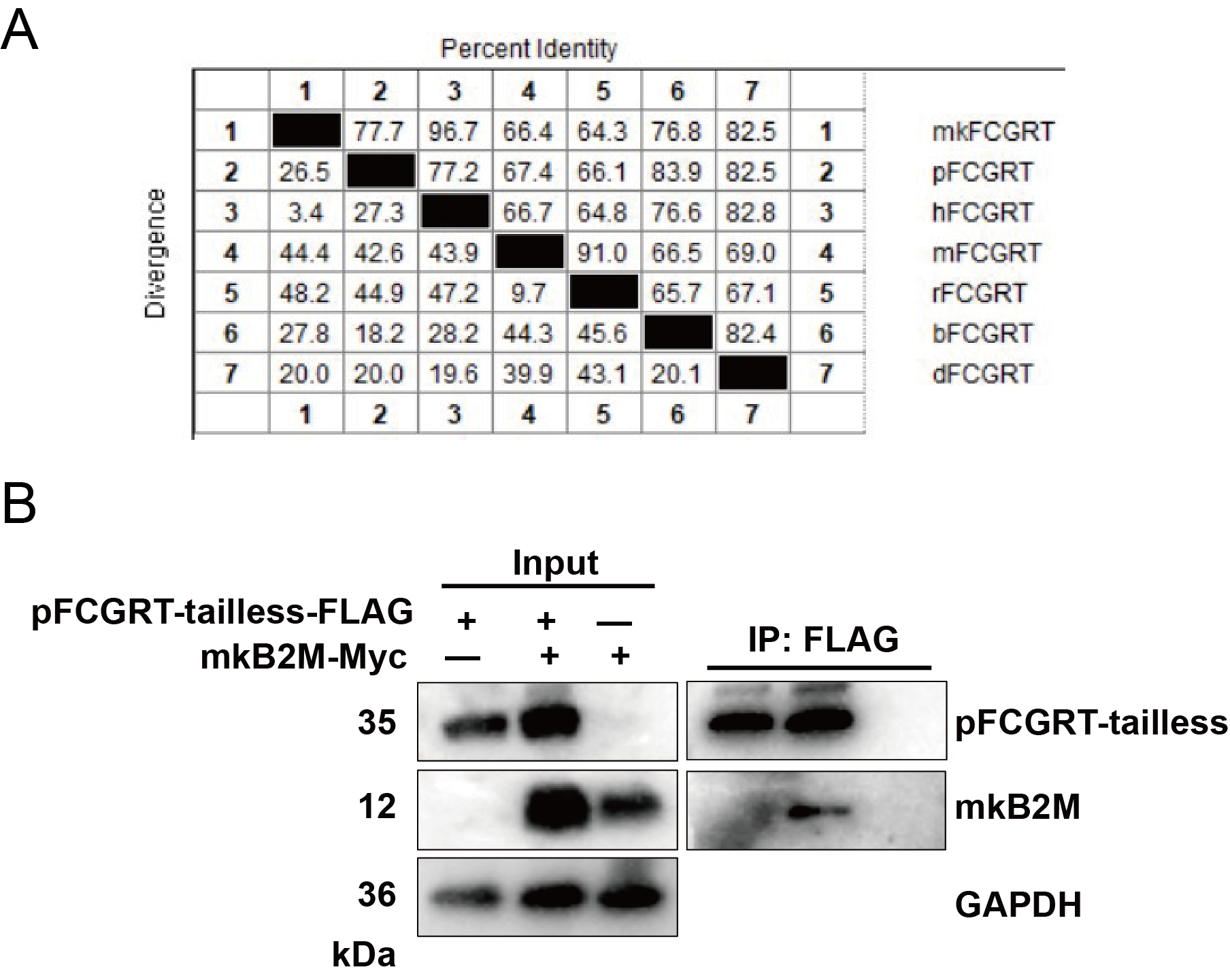


**Figure S4.** (A) The FCGRT from green monkey, pig, human, mouse, rat, bovine, and dog amino acid sequence were aligned using Megalign software, and percent identify were shown on the picture. (B) HEK-293T cells were cotransfected with expression vectors encoding FLAG-tagged pFCGRT-tailless and Myc-tagged mkB2M. The cells were lysed at 48 h posttransfection, and lysates were immunoprecipitated with anti-FLAG antibody. The whole cell lysates (input) and IP complexes were analyzed by Western blot with anti-FLAG, anti-Myc, and anti-GAPDH antibodies, respectively.


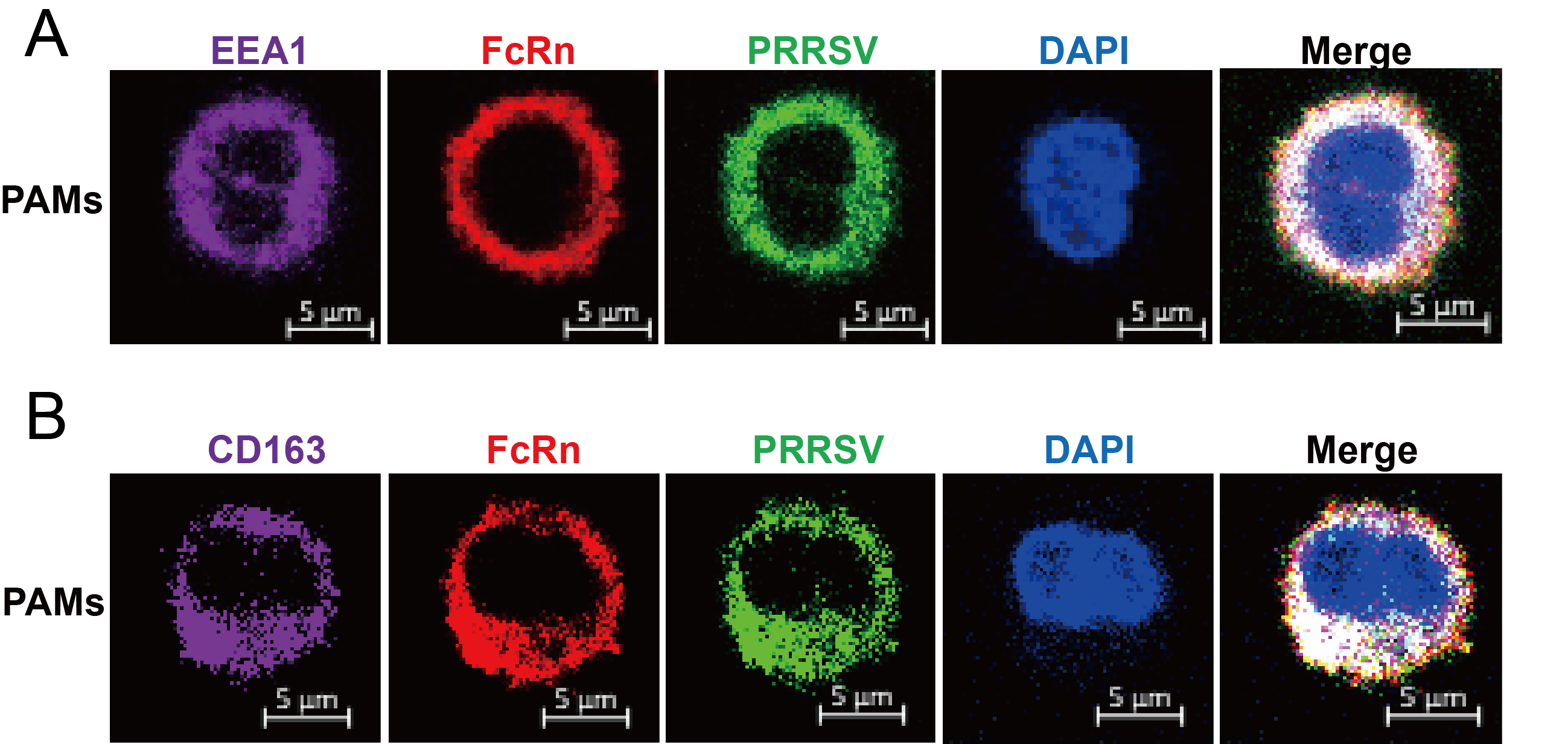


**Figure S5.** (A) PAMs seeded in confocal dish were prechilled at 4℃ for 1 h, and then the media were replaced by precooled DMEM containing PRRSV strain FJ (MOI of 1.0). After incubation at 37℃ for 30 min, the cells were first costained with mouse anti-EEA1 mAb (purple) and rabbit anti-pFCGRT-CT pAb (red), and pig anti-PRRSV pAb (green), respectively. After washes with PBS, the cells were costained with CoraLite647-conjugated goat anti-mouse IgG and ABflo®594-conjugated goat anti-rabbit IgG. After another washes with PBS, the cells were finally stained with FITC-conjugated rabbit anti-pig IgG and then detected using confocal microscopy. Nuclei were stained with DAPI (blue). Scale bars represent 5 *μ*m, and representative colocalization (white) images are shown. (B) PAMs were treated as described for panel A, but the EEA1 mAb was replaced with mouse anti-CD163 mAb. Scale bars represent 5 *μ*m, and representative colocalization (white) images are shown.


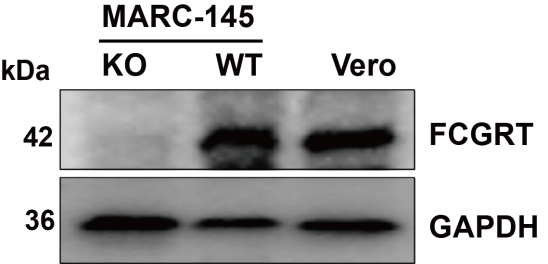


**Figure S6.** *FCGRT*-KO MARC-145, WT MARC-145, and Vero cells were cultured in 6-well plates until they reached full monolayer, and cells were harvested to detect the FCGRT expression by Western blot using anti-FCGRT pAb.
